# Empirical Support for the Involvement Load Hypothesis (ILH): A Systematic Review

**DOI:** 10.3390/bs12100354

**Published:** 2022-09-23

**Authors:** Sylvia Liu, Barry Lee Reynolds

**Affiliations:** 1 Faculty of Education, University of Macau, E33, Av. da Universidade, Taipa, Macau SAR, China; 2 Centre for Cognitive and Brain Sciences, University of Macau, Taipa, Macau SAR, China

**Keywords:** Involvement Load Hypothesis, vocabulary acquisition, aspects of knowing a word, task, systematic review

## Abstract

The Involvement Load Hypothesis (ILH) has become a widely used framework for predicting second language (L2) vocabulary learning from task completion. The purpose of this systematic review was to analyze the predictive ability of the ILH in the acquisition of aspects of knowing a word, its application in different target populations, the effective vocabulary learning task types designed based on the ILH, and the occurrence rate of the ILH components in vocabulary learning tasks. We searched IEEE, ERIC, WOS, Scopus, and ProQuest databases for empirical studies published between 2001 and 2021, using a vocabulary-focused keyword string combined with an ILH-focused keyword string. A total of 78 studies were selected using a set of inclusion and exclusion criteria. The content analysis of these studies showed that researchers have used the ILH to investigate the acquisition of six aspects of knowing a word. Four types of tasks (i.e., fill-in-the-blanks, reading, composition writing, and meaning-inferring) provided more positive evidence for the validation of the ILH. The search component was least present in the vocabulary learning tasks. Researchers have supported the use of the ILH to predict the vocabulary learning potential of tasks completed mainly by adult learners. This systematic review provides direction for future reviews and empirical studies in L2 vocabulary teaching and learning framed by the ILH.

## 1. Introduction

Word knowledge plays an unshakeable role in both foreign language (FL) and second language (L2) learning contexts [1]. Some learners have regarded vocabulary acquisition as their first priority and have felt that most of their challenges in communication have been due to inadequate vocabulary [2]. A very influential view of L2 vocabulary acquisition argues that the most important issue that learners face is expanding their vocabulary size [3], which has significant consequences on their later success and achievement in L2 learning [4]. As there is potential for great differences in the effectiveness of vocabulary learning activities, the importance of this issue has been emphasized by a large amount of research having been produced that investigates vocabulary task effectiveness. A possible explanation for the differences in task effectiveness is the amount of involvement that these tasks induce. For example, reading an article containing targeted words and then writing sentences with those targeted words might induce more effective vocabulary learning than reading an article containing targeted words and then copying those targeted words to a word list. This is because the first activity is more cognitively demanding. 

Researchers need to know how to measure and compare the effectiveness of vocabulary learning tasks. This is a complex issue to tackle in part because L2 learning experiences vary from learner to learner, with L2 learners engaging in a variety of different tasks throughout their learning journeys. The issue is further complicated by the fact that the complexities of knowing a word, the mode of learning (e.g., face-to-face or E-learning), and the use of different instructional strategies are only some of the influential factors that should be considered when deciding on whether one task is more effective than another. As a solution to this problem, initially developed and validated in 2001, the Involvement Load Hypothesis (ILH) has been considered as an easy-to-use predictive gauge of L2 vocabulary acquisition from completing different vocabulary tasks [3,5]. It provides researchers with a framework for predicting which tasks will be most effective in helping L2 learners to acquire new vocabulary. Although the ILH uses a simple and straightforward scale for calculating the involvement load, the ILH’s assumptions have not always been adhered to when it has been used by researchers for conducting empirical studies. Specifically, recent meta-analyses and conceptual works have reported contradictory findings regarding the predictability of the ILH from the studies reviewed and synthesized [6,7,8]. 

When first introduced, Laufer and Hulstijn [3] raised the importance of validating the ILH and using it as intended by adhering to its assumptions. Since 2001, the ILH has been widely adopted for over twenty years to assess the effectiveness of different vocabulary tasks. Unfortunately, many tasks that were purported to have been designed according to the ILH have sometimes been compared under heterogenous conditions. Ignoring the ILH assumptions will affect its predictive power. The current systematic review starts from the position that previous vocabulary task research should be re-examined in light of the assumptions proposed by Laufer and Hulstijn [3]. By reviewing studies from the past two decades that adopted the ILH as a theoretical framework, the present systematic review aimed to ascertain the extent to which the ILH has been supported by empirical evidence. Another aim of this research synthesis was to review the contexts and the quality of vocabulary learning tasks designed according to the ILH. 

## 2. Background Literature

### 2.1. The Basic Contention of the ILH

The ILH is charactered by its predictive ability of different tasks leading to second language (L2) vocabulary acquisition. It has played an important role in the study of L2 vocabulary learning for over two decades. The basic contention of the ILH is as follows:

Retention of unfamiliar words is, generally, conditional upon the degree of involvement in processing these words. In other words, it is conditional upon who has set the task, whether the new word has to be searched, and whether it has to be compared or combined with other words. The greater the involvement load, the better the retention [9], p. 545.

Involvement as it pertains to the ILH is defined as a motivational–cognitive construct [9]. The ILH contains three basic components: need, search, and evaluation. The need component is the only motivational dimension of involvement in the ILH. It refers to a task that is wanted (i.e., self-imposed) or required (i.e., externally imposed). When a learner is required to complete a task, the need is moderate. When a learner is intrinsically motivated to complete a task, the need is strong. The search and evaluation components belong to the cognitive dimension of involvement in the ILH. The search component refers to “the attempt to find the meaning of an unknown L2 word or the attempt to find the L2 word form expressing a concept (e.g., trying to find the L2 translation of an L1 word) by consulting a dictionary or another authority (e.g., a teacher)” [9], p. 544. The search component is either present or absent. The evaluation component refers to “assess[ing] whether a word does or does not fit its context” [9], p. 544. The evaluation component is moderate when a learner must compare an unknown L2 word with other words in a given context. The evaluation component is strong when a learner must use an unknown L2 word with other words in an original context. Moreover, any of the three components of the ILH can be absent. 

According to Hulstijn and Laufer [9], the involvement load (IL) is calculated as the sum of the three components’ degrees of prominence. The degree of prominence is described as an involvement index, which is shown by a number. For example, the absence of a component is given an involvement index of zero (i.e., index = 0), the moderate degree of prominence is given an involvement index of one (i.e., index = 1), and the strong degree of prominence is given an involvement index of two (i.e., index = 2). To operationalize IL, Hulstijn and Laufer [9] used two different tasks with two different ILs as examples. In the first task, L2 learners were asked to work on sentence-writing exercises using unknown words. Their teacher provided the L1 translations and explanations. According to the ILH, there was a moderate need as the task was externally imposed. The search component was absent as the word meanings and explanations were provided by the teacher. The degree of prominence in the evaluation component was strong because the L2 leaners had to use the new words together with suitable collocations in an original context. Hence, the IL of this task was 3 (i.e., IL = 1 + 0 + 2 = 3). In another task, the learners were asked to read a text with glosses and answer its accompanying comprehension questions. In order to answer these comprehension questions, the learners needed to know the meanings of the target words. The degree of prominence for the need component was moderate because the task was externally imposed. The search component was absent because the glosses of the target words were provided. The evaluation component was also absent because the context was provided to the learners. As a result, this task induced an IL of 1 (i.e., IL = 1 + 0 + 0 = 1). According to the basic contention of the ILH, Hulstijn and Laufer [9] concluded that task two was less effective, given that it induced a lower IL. 

### 2.2. Main Trends and Issues of the ILH

A small number of major trends and issues have been summarized in previous review and meta-analysis papers. Firstly, there has been a clear trend regarding the validation of the ILH: tasks that induced higher ILs led to more vocabulary acquisition [3,8,10,11,12]. For example, in San-Mateo-Valdehita’s review [12], the researcher analyzed a series of high IL writing tasks and other tasks with low ILs, concluding that writing was the most efficient task. However, a contradiction was evident in that some of the studies reviewed that did not show higher ILs also led to greater gains in vocabulary learning. In a more rigorous review, the included studies confirmed that higher ILs led to better vocabulary learning; however, the ILH explained a relatively low percentage in the overall learning gains [8]. 

Another trend has been to give each component of the ILH a different weight for the effect on L2 vocabulary acquisition. Back when the ILH was first proposed, Laufer and Hulstijn [3] used it to further analyze which tasks were more effective in 12 intervention studies published between 1992 and 1999, and found that these tasks induced at least one of the three ILH components. Specifically, compared to less efficient tasks, the high-efficiency tasks in six studies contained the evaluation component, the high-efficiency tasks in five studies contained the search component, and the high-efficiency tasks in one study contained both the need and search components. For the first time, they suggested that the three components might have different influences on L2 incidental vocabulary acquisition. A decade later, Boers and Lindstromberg [10] reviewed empirical studies published since 2004 that focused on evaluating the effectiveness of tasks for learning L2 formulaic sequences. Although the findings of their review showed “little evidence of the influence of this factor” [10], p. 100, the authors did not indicate how many studies this conclusion was based on. Recently, Yanagisawa and Webb [8] confirmed this statement in their meta-analysis, but the specific weight was slightly inconsistent with Laufer and Hulstijn [3]: the evaluation component had the greatest weight; the need component had a small weight; and the search component seemed dispensable.

In addition to the aforementioned two trends, there has been the argument that the low predictive ability and the unbalanced proponents also suggest other issues in addition to the ILH may impact vocabulary acquisition [8]. The early reviews and conceptual works mainly considered the following three issues: time-on-task, exposure frequency, and task types (e.g., [3,5]). As more relevant studies have been published, reviews in the last decade have covered the following issues: L2 proficiency level (e.g., [8]), multimedia presentation modes (e.g., [4]), task types (e.g., [13]), intentional learning (e.g., [14]), and generic approaches (i.e., positivism, interpretivism, and critical theory) (e.g., [15]).

Whilst the reviews and conceptual works have added valuable insights, they reviewed a limited scope of empirical studies. Given that tasks for L2 vocabulary acquisition could be highly varied due to the strong link with other issues, including the targeted population and aspects of knowing a word, there are a range of tasks that could be tailored to different learner populations [8]. The scope and classifications of L2 vocabulary acquisition of interest to the current systematic review, therefore, include aspects of knowing a word [1] that were not reported in previous reviews. Knowing a word is “a cumulative process involving a range of aspects of knowledge. Learners thus need many different kinds of meetings with words in order to learn them fully” [1], p. 4. To date, there has been little review on how tasks designed based on ILH contribute to the six aspects of knowing a word: receptive form, receptive meaning, receptive use, productive form, productive meaning, and productive use. To our knowledge, no systematic review has been conducted on the ILH in terms of these six aspects of knowing words. 

### 2.3. Research Aims and Questions of the Systematic Review

Our main aim was to identify, screen, and analyze as much research on the use of the ILH in L2 vocabulary learning as our resources would allow. We aimed to situate empirical studies investigating the prediction of the ILH on L2 vocabulary learning in terms of four aspects: word knowledge, task type, learning context, and ILH component (i.e., need, search, and evaluation) prevalence. Specifically, we aimed to answer the following research questions (RQs):

Which aspects of knowing a word have researchers used the ILH to investigate?

For which tasks have researchers used the ILH to assess their vocabulary learning potentials?

Which learner populations’ vocabulary learning potentials have been investigated by researchers using the ILH? 

Which ILH component (i.e., need, search, or evaluation) is most or least often present in vocabulary learning tasks used in the published literature?

## 3. Methods and Data Analysis

### 3.1. The Process of the Systematic Review

In linguistics and education literature, authors of previous systematic reviews have identified defining characteristics that differentiate the systematic review method from other qualitative review methods [16,17,18,19]. We have adapted these characteristics for the current systematic review:

The current review was conducted by two researchers.

We used transparent procedures based on the Preferred Reporting Items for Systematic Reviews and Meta Analysis (PRISMA) 2020 checklist [18].

We created a code book that specified how the studies were to be coded.

We applied five different sets of search strings for searching five well-constructed electronic databases. 

We attempted to reduce reviewer bias by double coding the studies and by providing quality evaluations of the studies reviewed.

We included unpublished studies (i.e., conference proceedings, theses, and dissertations).

### 3.2. Study Identification

#### 3.2.1. Database Searches

Five separate digital databases were searched in conformity with the PRISMA 2020 checklist and previous reviews [8,16,18]. The following databases were searched for relevant articles (e.g., [8,16]): Scopus, Web of Science (WoS), ProQuest Dissertation and Theses Global (PQDT), Institute of Electrical and Electronics Engineers (IEEE), and Education Resources Information Center (ERIC). The time span was from 2001 to 2021. The lower limit for the database searches’ beginning year was selected to reflect the time when the ILH was proposed by Hulstijn and Laufer [9]. Two search strings were used, each including search terms related to (1) ILH and (2) vocabulary. For example, we searched for studies that included [(“ILH” (Topic) or “involvement load” (Topic) or “task effectiveness” (Topic) or task-induced (Topic)) AND (word* (Topic) or vocab* (Topic) or collocation* (Topic) or “n gram” (Topic) or idiom* (Topic) or lex* (Topic) or chunk* (Topic) or phras* (Topic) or pattern* (Topic) or formulaic* (Topic) or figurative* (Topic) or fixed-frame* (Topic) or binomial* (Topic))] in the WoS.

To yield the best database search results, we then applied a comprehensive search strategy. Different search techniques were tested (e.g., the use of wildcards and/or limits of the specific database, Boolean operators, and the thesaurus) to ensure the best matches. These search techniques were refined by scrutinizing the titles retrieved and revising the combination of wild cards, search terms, or adding/deleting synonyms or limits. To increase the reliability, we conducted two separate rounds of searches and reached an excellent rate of agreement (i.e., 100%). Specific search strings of the other four databases can be seen in the Supplementary Materials (see Table S1).

#### 3.2.2. Screening

We then identified from titles and abstracts (and sometimes from reading the full texts) the studies to be selected for the current systematic review according to the following inclusion and exclusion criteria: 

#### 3.2.3. Included

Studies that reported on the ILH and L2 vocabulary learning.

Studies based on primary research data.

Studies published in book chapters (but not when duplicated in theses, dissertations, or journal articles).

Master and doctoral theses and dissertations.

Studies where the sample was selected at a pre-primary, primary, secondary, tertiary, or enrichment level. 

#### 3.2.4. Excluded 

Studies that did not mention the ILH.

Studies that reported on “task effectiveness” and/or “induced-task” in special needs education, neuroscience, and clinical research.

Review papers or conceptual works.

Studies that mentioned the ILH but did not test the prediction of the ILH or did not evaluate the factors that influence the prediction of the ILH.

Studies that reported research already published in other publications.

The database searches using key terms yielded 691 articles for possible selection. With the application of the manual-scan method, we removed 135 articles through the de-duplication process: (1) the 691 articles were sorted by title in EndNote 20; (2) the titles were scrutinized one by one with the aim of finding and eliminating duplicate titles; and, finally, (3) other aspects of the articles with the same titles (i.e., author, publication title, volume, and issue number) were verified to confirm a duplication. In collaboration with an expert external to the research team and through the application of the inclusion and exclusion criteria, we reduced the number from 556 to 119. We retrieved the full-text versions of these 119 articles and reapplied the inclusion and exclusion criteria. Two articles could not be extracted; one thesis/dissertation was under embargo, and one article could not be found in the journal to which the authors referred. After contacting the Scopus staff and the journal’s editor-in-chief, we concluded that this article had been erroneously indexed. The application of this in-depth screening finally yielded a total of 78 empirical studies. We calculated the inter-screener reliability using Cohen’s kappa and found a 99.86% agreement rate that was acceptable at *k* = 0.99. The only discrepancy was discussed and resolved. 

To sum up, regarding the study identification, quality control (QC) procedures were applied by a linear process: databases searched with key terms→de-duplicated→title and abstract screened→full-text extracted and securitized→selected 78 empirical studies, as the PRISMA flow diagram shows in the Supplementary Materials (See Figure S1).

### 3.3. In-Depth Data Extraction

#### 3.3.1. Extraction Procedure

For in-depth data extraction, we created a coding book in line with previous reviews. We piloted (*k* = 8 studies) and then updated the coding book to ensure all of the necessary information from each selected study could be extracted to answer the research questions of the current review (see Table S2 in Supplementary Materials). Two coders independently read each article thoroughly and coded the data by using NVivo 12 plus. In addition, both coders independently filled in a data extraction form. If data were not available in studies, we contacted the authors for additional information (*k* = 18). The two coders then compared their data extraction forms. After comparison, coding disagreements were resolved through consensus-oriented discussions and by consulting a third person. The detailed data extracted from the 78 studies are shown in the Supplementary Materials (See Table S3 and Table S4). 

#### 3.3.2. Quality and Relevance Appraisal

We adapted the Weight of Evidence (WoE) framework [16,20,21] of quality and relevance appraisal to judge the selected studies. The WoE accessed three aspects of empirical research: “soundness of studies, appropriateness of study design for answering the review question, and relevance of the study focus to the review” [21], p. 162. According to the WoE framework, each aspect was classified as high, medium, or low for each study. Examples of the specific scales for rating studies are shown in the Supplementary Materials (See Table S5). 

**Figure 1:**
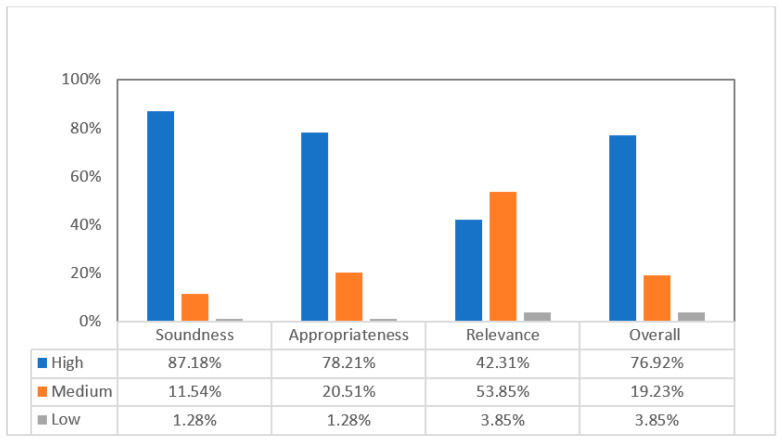
Distribution of the WoE Scores.

The overall WoE was calculated based on Harden and Gough [21], recommended method: For any selected study to be coded as high level, both soundness and appropriateness had to be coded high and relevance had to be at least medium. The 78 selected studies were judged independently by two researchers. We found a 98.08% agreement rate. Most of the articles (i.e., 96.15%) selected were in the medium- and high-quality range. Any discrepancies were discussed and resolved. Hence, a relatively accurate picture of the overall WoE was achieved. The WoE scores of the included studies are presented in Figure 1 below. 

Note: The qualities of the selected studies are shown for soundness, appropriateness, and relevance, and for low, medium, and high levels. The overall scores were calculated according to Harden and Gough’s [21] recommended method.

Figure 1 shows that, of the 78 studies, 87.18% were judged as having a high level of soundness, 11.54% a medium level, and only 1.28% a low level of soundness. This shows that the overall trustworthiness of the selected studies in this review is very high. Only 1.28% of the selected studies adopted research methods that we judged as having low appropriateness, and 20.51% as fairly appropriate. Most of the studies (i.e., 78.21%) were judged as highly appropriate for answering the current systematic review RQs. Regarding relevance, 42.31% studies were judged at high level and 53.85% at medium level, leaving only 3.85% at low level. This indicated that the research topics of the selected studies were indeed of interest to this review. Hence, we concluded that 96.15% of the 78 studies were able to make a strong contribution to this review’s research questions. 

#### 3.3.3. Coding

To guide our initial review and begin answering our review questions, we first relied on *EndNote 20* to help us select and categorize studies obtained through electronic database searches. Next, we used the *NVivo 12* to code the selected studies. During the coding process, we first assigned codes for the following characteristics of each study: demographic information of the empirical study (e.g., publication title, year, and country); demographic information of participants (e.g., educational level, L1 backgrounds, and L2); research methods, tasks, research sites (e.g., classrooms, laboratories, online, and home); and vocabulary acquisition measurements. The specificity of the codes was checked against the following resources: previously published reviews and meta-analysis research (e.g., [6,7,8,22]); *The Literacy Dictionary* [23]; and the related theoretical works in L2 vocabulary acquisition (e.g., [1,3,5,24]). Furthermore, to access the coding reliability, two coders coded the studies individually. The average agreement was 96.15%, showing satisfactory agreement across the codes. In addition, the mixed method studies that included qualitative components were coded with the same set codes, except for hypothesis and effect size.

## 4. Results

### 4.1. General Description of Included Studies

#### 4.1.1. Publications

The analyzed sample is composed of 63 journal articles, 4 conference papers, and 11 theses. Among these, three journals published four or more articles, namely, *Language Teaching Research*, *International Review of Applied Linguistics in Language Teaching (IRAL)*, and *System*. Most of these are first-quartile (Q1) journal articles under the scope of linguistics and/or education and educational research according to Journal Citation Report (JCR) 2022′s Journal Citation Indicator (JCI) rankings for 2021 (see Table 1).

**Figure 2:**
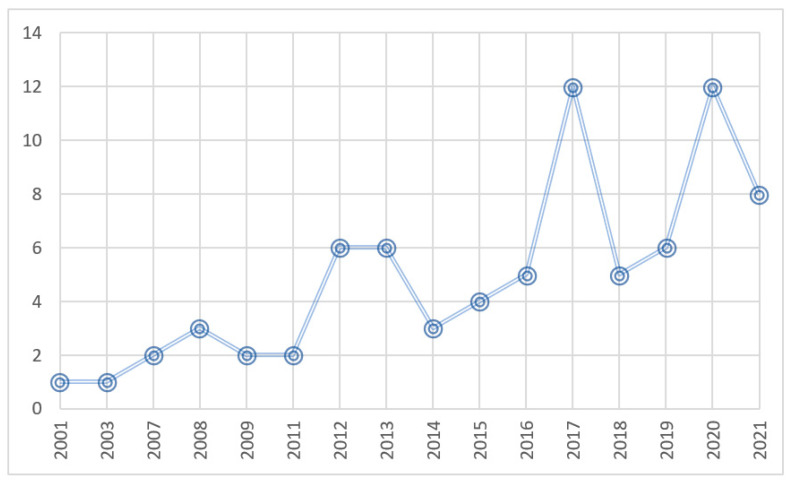
Number of publications per year.

Studies about the ILH and L2 vocabulary learning are steadily growing, with an increase of 23.78% studies per year. As shown in Figure 2, the majority of studies have been conducted from 2017 onward, increasing by more than one-half (*k* = 43) of the total studies (*k* = 78). This indicates that the ILH is a rapidly growing research topic. 

#### 4.1.2. Countries

**Figure 3:**
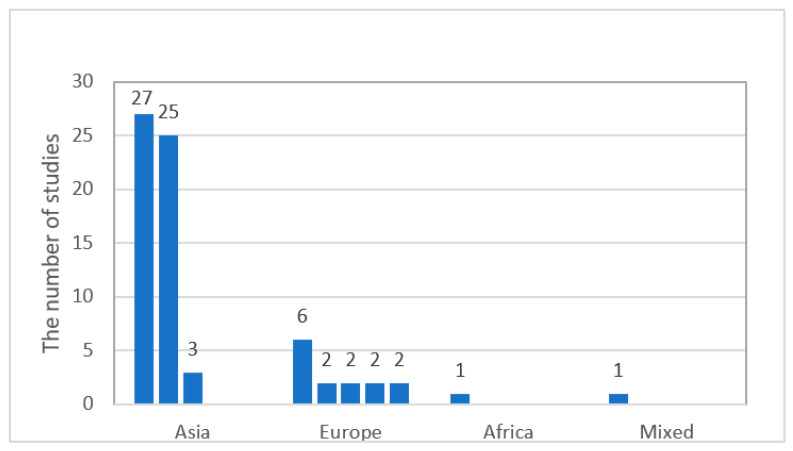
Geographical distribution.

Based on our findings, the ILH as related to vocabulary learning had been studied across a broad geographic area (Figure 3). The 78 studies we selected were carried out in 26 countries from three continents: Eurasia (88.46%, *n* = 69) [i.e., Asia (70.51%, *k* = 55), Europe (17.95%, *k* = 14)], North America (8.97%, *k* = 7), and Africa (1.28%, *k* = 1). One study was classified separately as “mixed” because ILH tasks were completed online, and participants were from all over the world. Most of the studies were from Asia, particularly West Asia (31.62%, *k* = 27,) and East Asia (32.05%, *k* = 25). Southeast Asian countries were the least represented (3.85%, *k* = 3). Interestingly, there were no such studies in Central or South Asia. Iran (25.64%, *k* = 20), Greater China (23.08%, *k* = 18), and the United States (5.13%, *k* = 4) had the most ILH publications. The 14 studies conducted in Europe were relatively evenly distributed across regions of Europe, except for Western Europe (7.69% *k* = 6). There were two studies (2.56%) each in Southern Europe, Central Europe, Eastern Europe, and Northern Europe. All seven (8.97%) of the North American studies were conducted in either the United States (5.13%, *k* = 4) or Canada (3.85%, *k* = 3). The only African study came from Egypt (1.28%, *k* = 1).

#### 4.1.3. Languages 

**Figure 4:**
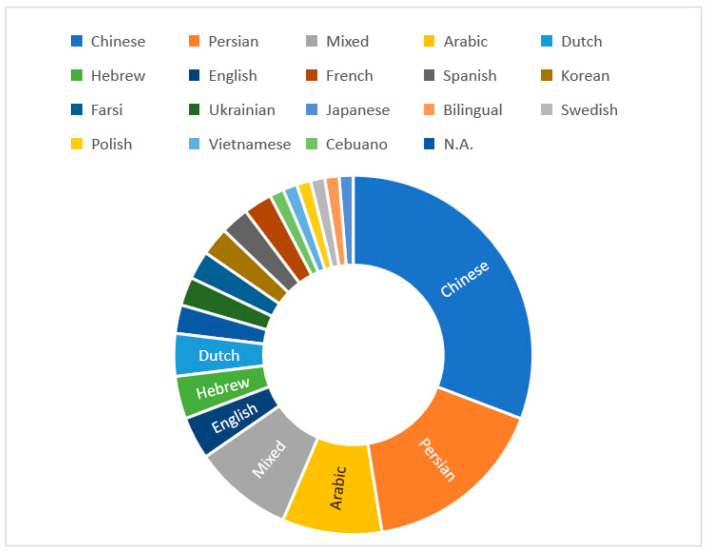
The L1 Backgrounds.

Among the 78 studies included in the analysis, participants spoke 17 different L1s, with Chinese (30.77% *k* = 24), Persian (16.67%, *k* = 13), and Arabic (8.97%, *k* = 7) being the top three (see Figure 4). The L1 analysis also showed a prevalence of mixed L1 backgrounds (8.97%, *k* = 7). Moreover, two studies were coded as “not available (N.A.)” because the original authors did not provide this information.

**Figure 5:**
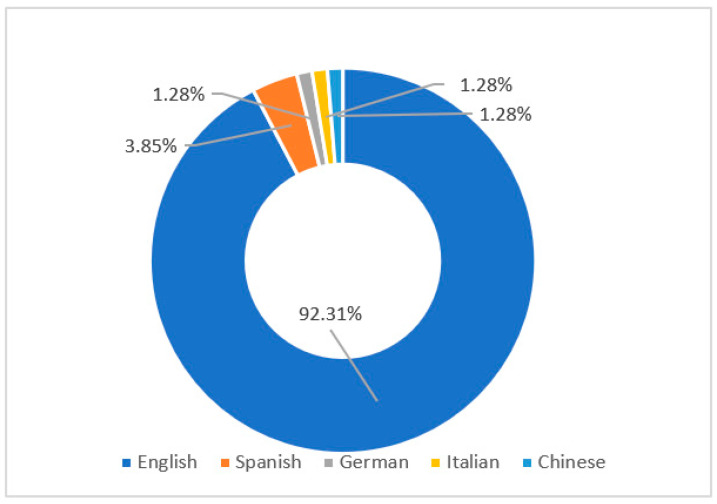
The targeted L2s.

The same simple statistical analysis was used to analyze the targeted L2s. Figure 5 shows an overview of the targeted languages of the 78 selected studies. We found that the ILH was not only applied to the vocabulary acquisition of English as a Second Language (ESL) or foreign language (EFL), but was also applied to other languages learned as L2s. Although most of the selected studies focused on English (92.31%, *k* = 72), three studies (3.85%) focused on Spanish, one (1.28%) focused on German, one (1.28%) focused on Italian, and one (1.28%) focused on Chinese. 

### 4.2. Review RQ1: Which Aspects of Knowing a Word Have Researchers Used the ILH to Investigate?

#### Number of Aspects of Knowing a Word Assessed

**Figure 6:**
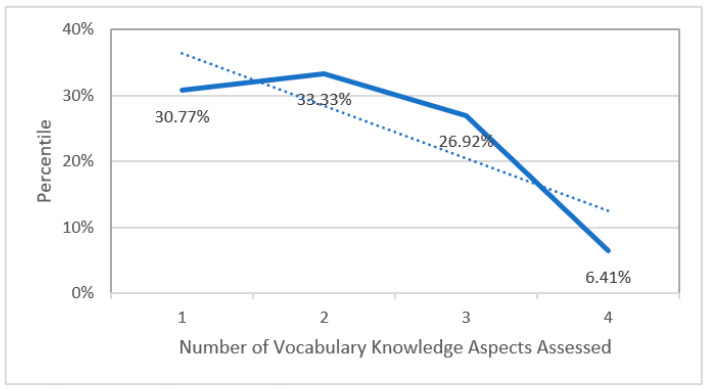
Number of aspects of knowing a word assessed per study.

As shown in Figure 6, we found a linear trend: 24 (30.77%) studies focused on the effect of vocabulary learning tasks on one aspect of knowing a word, 26 (33.33%) on two aspects, 21 (26.92%) on three aspects, and only 5 (6.41%) on four aspects. 

This indicated that tasks based on the ILH only included up to four aspects of knowing a word; none of the studies included a task where five or all six aspects of knowing a word were assessed. Moreover, two studies (2.56%) were coded as “N.A.” because the information was not provided by their original researchers.

**Figure 7:**
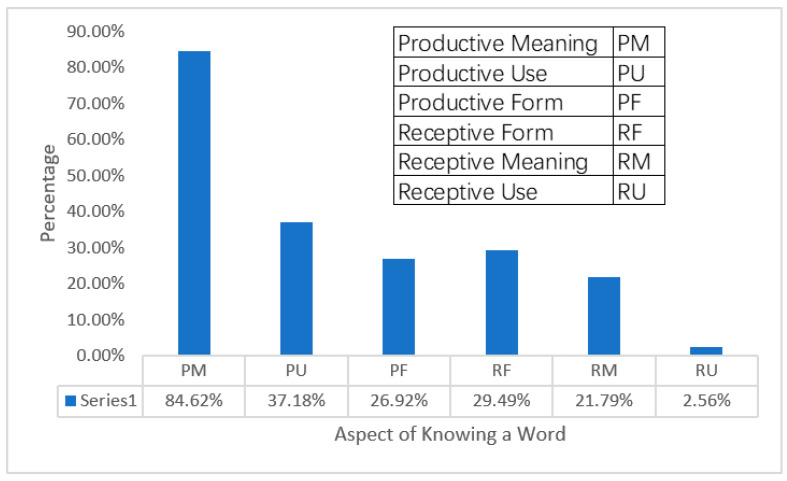
Aspects of knowing a word.

Regarding which aspects of knowing a word researchers used the ILH to investigate, we found it interesting that researchers preferred to investigate the productive rather than receptive aspects of knowing a word (see Figure 7). 

Further analysis of the individual aspects of knowing a word, divided into productive and receptive categories, showed that the majority of the selected studies (94.87%, *k* = 74) investigated the prediction of the ILH on the productive category of knowing a word, with productive meaning (PM) (84.62%, *k* = 66) being the top focus. Furthermore, 29 (37.18%) studies focused on testing the predictive power of the ILH in the productive use (PU) aspect, and 21 (26.92%) studies focused on the productive form (PF) aspect. Regarding the receptive aspects of knowing a word, 23 (29.49%) studies focused on testing the predictive power of the ILH in the receptive form (RF) aspect, 17 (21.79%) on the receptive meaning (RM) aspect, and 2 (2.56%) on the receptive use (RU) aspect. Overall, these results indicated that researchers have used the ILH to investigate the acquisition of all six aspects of knowing a word, with PM, PU, and RF being the top three aspects. 

### 4.3. Review RQ2: For Which Tasks Have Researchers Used the ILH to Assess Their Vocabulary Learning Potentials?

**Figure 8:**
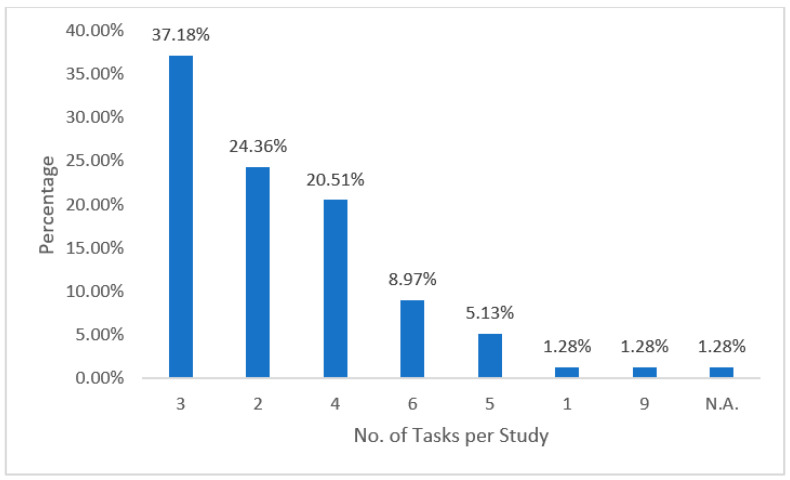
Tasks per study.

In total, there were 262 tasks in the 78 selected studies. As shown in Figure 8 below, in most studies, researchers tended to evaluate several different tasks in a single study.

Their most common practice was to compare and evaluate two to four different tasks in one study. One interesting finding was a single study (1.28%, *k* = 1) in which researchers compared and evaluated nine different learning tasks [25]. In another study (1.28%, *k* = 1), researchers gave an in-depth evaluation of just one task [26]. A further 24.36% of the studies included two different tasks, 37.18% included three different tasks, 20.51% included four different tasks, 5.13% included five different tasks, and 8.97% included six different tasks. In addition, in one study (1.28%, *k* = 1), the researchers did not describe the task. 

**Figure 9:**
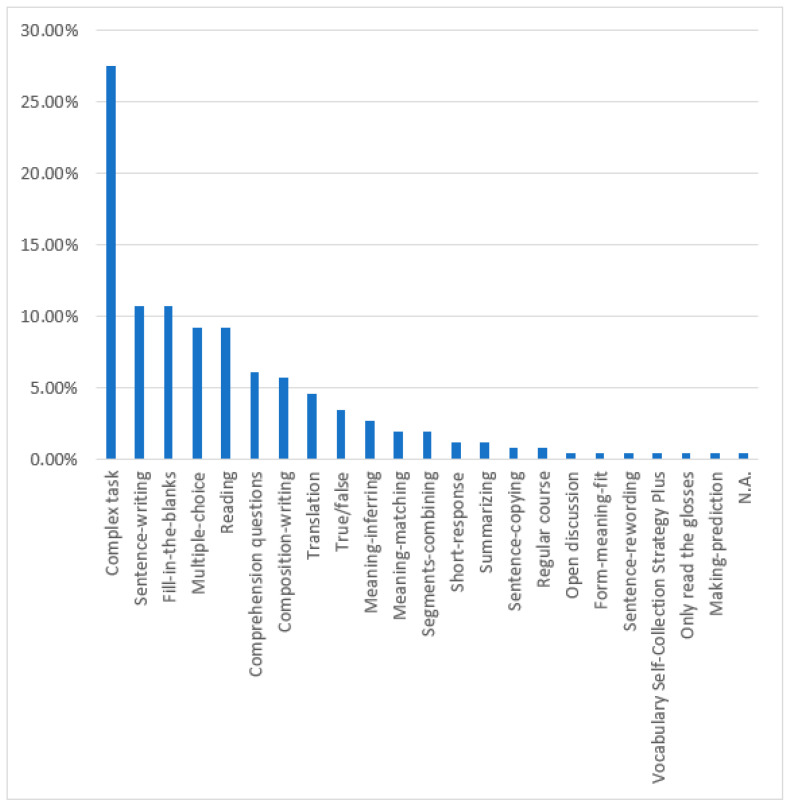
Task types.

We also found a very interesting phenomenon. Among the 78 studies, there were 22 unique types of L2 vocabulary learning tasks designed by researchers according to the ILH (see Figure 9 and Table 2). This finding was reached through two rigorous steps. We first extracted and carefully reviewed the ILH-based tasks described in each study. We then classified and summarized the characteristics of each task by using the in vivo coding method [27].

We divided the 22 different task types into three categories according to the number of times that they had been investigated in the 78 studies. They were categorized as high-frequency tasks (>20 of the 78 studies), medium-frequency tasks (<20 and >5 of the 78 studies), and low-frequency tasks (<5 of the 78 studies). 

**Figure 10:**
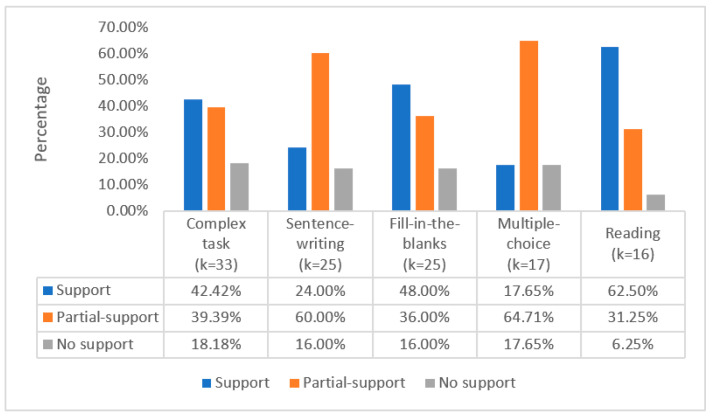
High-frequency task types and ILH support in studies.

The most frequent was the complex task type, combining several individual tasks into one task (See Figure 10). A total of 72 different complex tasks were used in 33 studies. Most of the studies showed that complex tasks support (42.42%, *k* = 14) or partially support (39.39%, *k* = 13) the predictability of the ILH. Although 81.82% of the 33 studies showed how their findings provided support to the predictions of the ILH, 18.18% (*k* = 6) of the studies found that the predictions were not supported. The next most common high-frequency task types included: sentence-writing, fill-in-the-blanks, multiple-choice, and reading. A total of 28 different sentence-writing tasks were used in 25 studies. Most of the studies showed that sentence-writing tasks provided at least some support for the predictability of the ILH. In contrast, four studies (16%) showed that learners in the high-IL task groups did not learn the L2 vocabulary more effectively than learners in the low-IL task groups. Likewise, a total of 28 different fill-in-the-blank tasks were used in 25 studies. Most of the studies showed that fill-in-the-blank tasks support (48.00%, *k* = 12) or partially support (36.00%, *k* = 9) the predictability of the ILH. Again, four studies (16%) reported contradictory results. Seventeen studies included at least a multiple-choice task, similar to the sentence-writing tasks, with more studies (64.71% *k* = 11) partially supporting the ILH than fully supporting the ILH (17.65%, *k* = 3). In addition, three studies (17.65%) did not support the ILH. Of the 16 studies that included a reading task, 10 studies (62.50%) confirmed the ILH’s prediction, 5 studies (31.25%) provided partial evidence for it, and 1 study (6.25%) failed to support it. 

**Figure 11:**
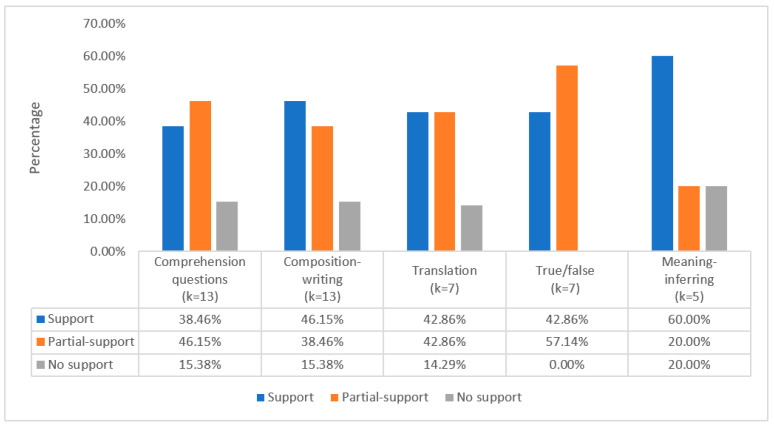
Medium-frequency task types and ILH support in studies.

The medium-frequency task types included five task types: comprehension questions, composition writing, translation, true/false, and meaning-inferring. Most studies involving these types of learning tasks provided solid supporting evidence for the ILH (see Figure 11). A total of 16 different comprehension question tasks were used in 13 studies, with 5 studies (38.46%, *k* = 5) supporting the ILH, 6 studies (46.15%, *k* = 6) partially supporting the ILH, and 2 studies (15.35%, *k* = 2) not supporting the ILH. Meanwhile, the composition-writing tasks also showed very similar results, except that the number of studies partially and fully supporting the ILH was reversed. A total of 12 translation tasks were used in seven studies. Three studies (42.86%) confirmed the ILH’s prediction, three (42.86%) partially confirmed it, and one (14.20%) did not support the ILH. Interestingly, although there was only a small number of true/false tasks (*k* = 9) used for seven studies, all of those studies provided positive evidence for the ILH predictions. For the meaning-inferring tasks, a total of seven tasks were used in five studies. Unlike the previous task types, only 80% (*k* = 4) of meaning-inferring tasks provided positive support for the predictability of the ILH.

**Figure 12:**
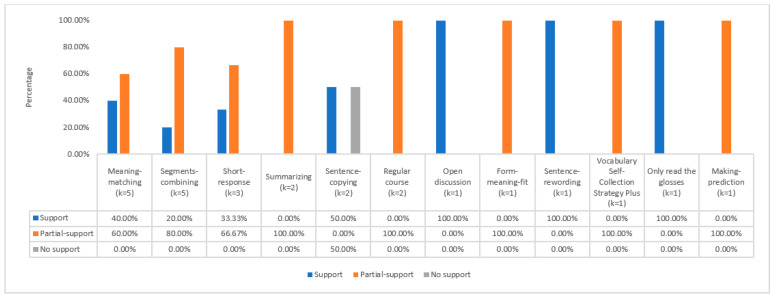
Low-frequency task types and ILH support in studies.

The low-frequency task types occurred between one and five times in the reviewed studies (see Figure 12). In short, this group contained twelve types of tasks. Five meaning-matching tasks were found in five studies, five segment-combining tasks in five studies, three short-response tasks in three studies, three summarizing tasks in two studies, and two sentence-copying tasks in two studies. Moreover, two studies had two regular courses as their control tasks. Researchers evaluating the ILH’s predictive abilities found that five task types provided positive evidence, but sentence-copying yielded ambiguous results (see Figure 12). The remaining task types only appeared once. The six rare task types were open discussion, form-meaning-fit, sentence-rewording, The Vocabulary Self-Collection Strategy Plus, only reading the glosses, and making a prediction. Overall, the six task types provided positive evidence for the ILH, with the sentence-rewording task and only reading the glosses task providing comprehensive support for the ILH. 

After a comprehensive analysis based on the main results of each study and the tasks chosen by the researchers, we found that four types of tasks provided more positive evidence for the validation of the ILH. The four types of tasks were fill-in-the-blanks, reading, composition writing, and meaning-inferring. Studies involving these four types of L2 vocabulary learning tasks frequently proved the predictive ability of the ILH. Although the complex task type also provided positive evidence for the ILH, it was a very complex system to analyze; this will be covered later in the discussion. 

### 4.4. RQ 3: Which Learner Populations’ Vocabulary Learning Potentials Have Been Investigated by Researchers Using the ILH?

To fully answer this review question, we first analyzed the L2 learning environment in each study. According to our preliminary analysis, most studies (96.51%, *k* = 75) investigated the vocabulary learning potentials of the participants in the foreign language learning context, and only a few studies (3.85%, *k* = 3) investigated the vocabulary learning potentials of the participants in the second language learning context. As for the specific research site of each study, sixty-four studies (82.05%) were conducted in classrooms, three (3.85%) in linguistics laboratories, two (2.56%) online, two (2.56%) in composite research sites (i.e., classroom + home and classroom + laboratory), and one (1.28%) in a school computer area. In addition, the researchers of six (7.69%) studies did not state where the studies were conducted. 

**Figure 13:**
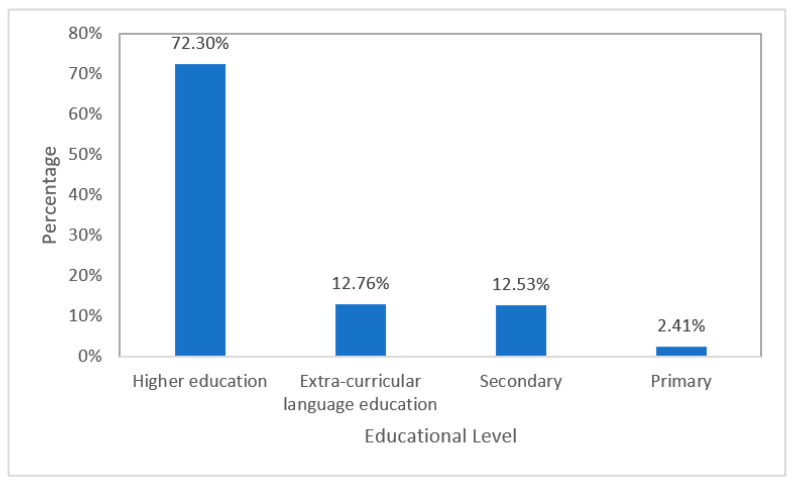
Educational level.

Further analysis showed that the participants in these studies varied in education level and age. The 78 studies involved a total of 6805 participants, including 4920 (72.30%) from higher education, 868 (12.76%) from extra-curricular language education, 853 (12.53%) from secondary education, and 164 (2.41%) from primary education. This suggests that ILH research in primary education is still in its infancy (see Figure 13 below).

Most research took place at the higher education level. For example, 53 studies (67.95%) recruited university students as the target study sample. These participants ranged in age from young adults to middle-aged adults. Fourteen studies focused on extra-curricular language education, accounting for 17.95% of the total studies reviewed. With teenagers to middle-aged adults, the age range of participants in extra-curricular studies was broader than that of the higher education participants. Moreover, only a few researchers focused on primary and secondary education levels. There were six studies (7.69%) investigating the effectiveness of tasks on vocabulary learning among secondary school students (i.e., teenagers). Only five studies (6.41%) investigated the effects of different learning tasks on primary pupils’ L2 vocabulary acquisition. In short, a great deal of the existing research has focused on adult populations, but relatively little research has been conducted on young children. 

### 4.5. RQ 4: Which ILH Component (i.e., Need, Search, or Evaluation) Is Most or Least Often Present in Vocabulary Learning Tasks Used in the Published Literature? 

**Figure 14:**
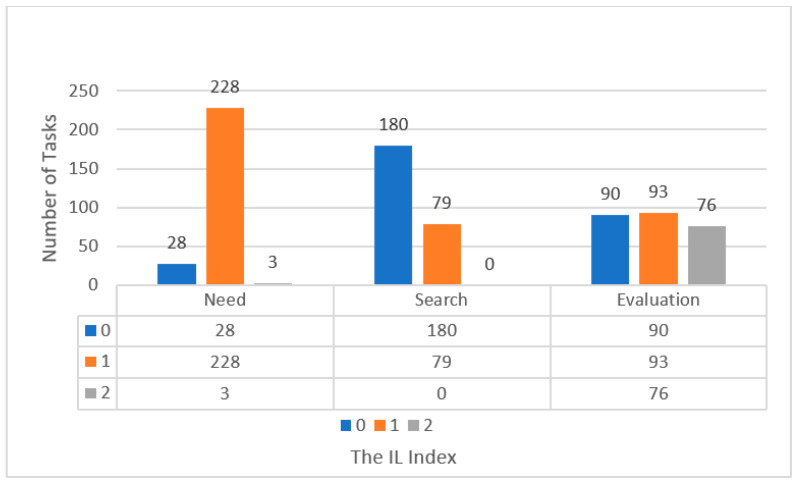
The ILH components. Note: While the total number of tasks reported in the studies equaled 262, the maximum number in this figure is 259. This is because the descriptions of three tasks in the published research did not provide enough information for coding.

In this systematic review, we recalculated the IL index of each vocabulary learning task strictly according to Laufer and Hulstijn’s [3] initial description of the ILH. We adjusted the ILs of the tasks in more than half of the 78 studies because we found that the ILs of many so-called L2 vocabulary learning tasks were not calculated according to the original ILH. This issue is consistent with the previous meta-analysis of Yanagisawa and Webb [8]. Hence, we recalculated the tasks of 20 studies. Moreover, we calculated the tasks involved in 22 studies in which researchers did not code the IL of their tasks. In summary, 53.85% (*k* = 42) of the 78 studies’ vocabulary learning tasks had to first be updated according to the original ILH. Then, the ILH components included in each individual L2 vocabulary learning task could be extracted. In the 262 different tasks, we found that the most common ILH component presented in vocabulary learning tasks was need, and the least often presented was search (see Figure 14 below). 

It is worth mentioning that there were three special tasks, among which, the need components were coded as strong, which means that the participants were internally motivated to complete the three vocabulary learning tasks.

## 5. Discussion

The review provided some theoretical and practical support for the ILH. The results showed that vocabulary learning tasks designed according to the ILH have been investigated for all six aspects of knowing a word. When we re-examined the 78 empirical studies using Nation’s [1] terminology for knowing a word and the original ILH, we found positive evidence for the ILH predictive ability for vocabulary acquisition. However, we also found that the effectiveness of tasks under the same type of vocabulary learning activities could vary across studies, as many studies that reported L2 vocabulary learning tasks designed according to the ILH were not as effective as predicted by the ILH. In some studies, only some of the results were consistent with the ILH predictions, whereas, in others, all of the results were completely contrary to ILH predictions. Hence, the predictive power of the ILH was greatly reduced. This finding agrees with Yanagisawa and Webb’s meta-analysis findings [8], which showed that the predictive power of the ILH is not very strong. One possible reason for this inconsistent predictive power of the ILH is that, even though the same task types are used in the studies, the measurement tools used to assess the aspects of knowing a word are different. For example, in Kaivanpanah et al.’s study [28], the IL was three for both the reading + multiple choice + reference dictionary task and the composition-writing task. According to the original assumption of the ILH, both tasks are equally effective for L2 vocabulary acquisition. However, Kaivanpanah et al. [28] found that participants in a composition-writing task group scored significantly higher on the knowing a word measurement test than the other task group. In this study, the measurement tool used aimed to measure productive aspects of knowing a word (e.g., productive meaning); however, the reading + multiple choice + reference dictionary task’s emphasis is on receptive aspects of knowing a word (e.g., RM). If they had chosen a vocabulary measurement that focused on the receptive meaning aspects of knowing a word, the results would have been very different. The reason may be related to the measurement tool chosen for their study. We found this issue to be common in many studies. This suggests that ILH’s inconsistent prediction of L2 vocabulary learning may be caused by an inconsistent choice of tasks and assessment tools (i.e., aspect of word knowledge assessed).

Whilst we fully acknowledge the importance of exploring the empirical support of the ILH on L2 learners’ vocabulary acquisition, a critical issue arises when we look at the bulk of the research on the aspects of knowing a word. This issue is about the selection of vocabulary measurement tools. The most common vocabulary measurement tools used in the 78 selected studies were designed by the researcher to assess one to four aspects of knowing a word, although a few studies adopted standardized vocabulary tests to examine learners’ learning outcomes on the RM, PM, and PU aspects of knowing a word. As we have noted, the current systematic review also revealed some inconsistencies in the aspects of knowing a word targeted for specific tasks and the aspects of knowing a word assessed by the measurement tools. On one hand, this inconsistency may be due to the researchers’ different understandings of both the three components of the ILH and Nation’s terminology [1] for knowing a word. On the other hand, this might also reflect the difficulty of designing measurement tools matched to the specific vocabulary learning tasks that researchers have designed. That is, designing measurement tools to cater to all of the vocabulary learning tasks in one study might sometimes be difficult. We encourage future researchers that specially design vocabulary measurement instruments for their studies to ensure that there is consistency between the aspects of knowing a word required to be understood or produced by learners and the aspects of knowing a word that are measured with any vocabulary measurement instruments. For example, some possible vocabulary measurements are word form recognition, word meaning recognition, and word use recognition (e.g., [1,22,24]). These tests can take several formats, such as true/false, multiple-choice, and matching the L2 synonyms or matching the L1 translations. The productive aspects of knowing a word can be assessed through vocabulary production tests such as translation and writing (e.g., sentence-writing). 

Previous review studies focused on improving the ILH by targeting potential confounding factors (e.g., time-on-tasks, frequency of exposure, and weight of the ILH components). Therefore, complex task types may not have fallen within the confines of their selection criteria. For example, the following types of studies have been excluded from previous meta-analyses: studies of tasks based on ILH design in the context of intentional learning, studies of “deliberate vocabulary learning activities” or “multiple language tasks” based on the ILH, and studies that did not calculate the ILs of the tasks (e.g., [7,8]). In addition, in the review by Hazrat and Read [6], they did not report either how many studies they reviewed or their study selection criteria. As a result, a portion of the empirical studies in the L2 vocabulary acquisition literature were likely selectively omitted by the researchers. However, since the ILH was first proposed, Laufer and Hulstijn [3] have emphasized the importance of task types in L2 vocabulary learning. They have suggested that one dimension for future research is examining the effects of task type with regard to the predictive ability of the ILH. As one of the characteristics of systematic review studies is comprehensiveness, we have filled in this gap by reviewing L2 vocabulary learning tasks in a wider range of studies than in previous reviews and meta-analyses. 

The results accrued from analyzing a total of 262 different vocabulary learning tasks seem to suggest that the ILH has been investigated with a variety of L2 vocabulary learning tasks. We have summarized 22 task types from 216 individual tasks for the first time, and many of the complex tasks were first synthesized in the present study. To our knowledge, while many complex tasks have emerged in recent years, combining multiple different small tasks to meet the needs of the L2 vocabulary teaching and learning in real classroom contexts, our review is the first attempt to comprehensively analyze and summarize these tasks according to the original ILH. We found the ILH to have stronger potential as a learning task design tool for predicting L2 vocabulary acquisition than previous review research has suggested. According to the ILH, L2 vocabulary acquisition is usually promoted by learners completing specially designed vocabulary learning tasks. Our results also suggest that the ideal specially designed tasks of the complex task type have the following characteristics: they must be interesting, relevant to the target second language vocabulary, and must provide adequate input. Future research can consider adjusting components of the complex task according to the targeted population’s learning needs, which can greatly improve the effect of vocabulary learning. 

Additionally, when we looked closely at individual studies and compared the different participant groups in each study, we often found factors other than the ILH components unequal. A common feature we found in these studies was that participants within a given study varied in age, L2 proficiency, and time-on-task. This finding suggests that the criticisms levied against the ILH in previous research may not have been justified. If, in a study, researchers had recruited participants of the same age with similar L2 proficiency to complete these complex tasks, the results might have been quite different. This again corroborates the second assumption of the ILH that “Other factors being equal, words which are processed with higher involvement load will be retained better than words which are processed with lower involvement load” [3], p. 15). Practically, in a real-life situation, every target word has the potential to have a different IL. However, when conducting research, we must select target words that are similar and with group comparisons using samples that are the same.

In addition to the complex task type, we also found that several task types were more consistent with the prediction of the ILH than others. Those task types are reading, fill-in-the-blanks, composition writing, and meaning-inferring. As mentioned in the Results section above, the predictive ability of the ILH was largely validated in studies that designed at least one of these four task types, as we found that most of the experimental results provided at least partial supporting evidence. These results suggest that we can give priority to these task types (i.e., reading, fill-in-the-blanks, composition writing, and meaning-inferring) when designing learning tasks in practice, thus improving the efficiency of L2 vocabulary learning. Furthermore, these four different task types have been studied many times in the past and have been applied to different L2 language levels, different learning contents, and different learning contexts. Hence, they have laid a foundation for future research.

The third research question of this review focused on the targeted population. The findings showed that adult learners have become the main population studied. According to our analysis, the vast majority of these adult learners have received higher education, and, as such, their cognitive abilities and various learning skills have been relatively mature. Thus, relatively speaking, learning tasks with high IL may not truly be high for this learner population. This explains why a large number of studies have partially supported the ILH, especially those in which many tasks with an IL difference of less than 2 showed no learning difference. Furthermore, some studies used secondary school students as their samples. An interesting research finding is that some researchers have applied the ILH to intentional vocabulary learning (e.g., [29,30]). Although the results of these studies provide only a small amount of support for the ILH, they also provide a new direction for future research because the implementation of incidental L2 vocabulary learning in secondary education, especially in the L2 classrooms, is feasible in theory, but difficult in practice. Hence, future studies may consider how to design L2 vocabulary learning tasks suitable for secondary school students by combining theory with practice and the task types recommended in answer to our second research question. In our view, designing the most suitable L2 vocabulary learning task for learners requires a review of previous successful cases to determine how the task meets learners’ learning preferences, learning needs, and development levels.

Another important finding of this study was that only 164 (2.41%) of the participants were primary school students, and none of the participants were younger than primary school age. This finding suggests a research gap: although the ILH has existed for more than 20 years, its application to children’s L2 vocabulary acquisition has been virtually nil due to the low number of studies found (i.e., *k* = 5) and the low WoE value of these five studies. For example, in Arabiana et al. [31], the following problems emerged: the task description was not clear enough to calculate the IL; the researchers did not report how the vocabulary acquisition was measured and scored; the researchers did not mention the duration of the intervention; and the researchers did not mention where the research was conducted (for example, in a classroom, online, or at the student’s home).

Moreover, we recalculated 55.85% (*k* = 42) of the 78 studies’ L2 vocabulary tasks based on the original ILH assumptions. As a result, we found search to be the least frequent ILH component. The majority of tasks presented unfamiliar words in glosses, saving participants the time of looking up unfamiliar word forms or meanings. In a few studies, teachers or peers explained the forms or meanings of unfamiliar words to the participants. This finding suggests that the statement that the three components of the ILH may have different proportions may be biased because the search component itself has been studied much less than the other two components. 

## 6. Implications and Limitations

### 6.1. Further Investigation into the L2 Vocabulary Acquisition

First, future meta-analysists could consider conducting a review of only studies that include tasks with the search component. This may overturn the previous argument that the three components of the ILH may carry different weights within a single task. Furthermore, as we mentioned earlier, most of the studies used a sample of adult learners. In this digital age, almost everyone has a smartphone with an electronic dictionary app. The traditional dictionary has begun to be phased out in classrooms. Therefore, when designing an L2 vocabulary task, we can properly consider the current digital learning mode and integrate the search component within electronic apps to advance with the times. 

Second, future review studies could compare the effectiveness of the ILH tasks at the same educational level because students of different education levels have many different factors, such as age and the level of development. Furthermore, future empirical studies may need to explore the predictive ability of the ILH on L2 vocabulary acquisition in primary schools or kindergartens. From the results of our review, empirical studies on the ILH at these two levels of education are lacking. For example, research is needed to show whether a high IL task is realistic for young learners in primary school or the preschool stage of education. The long-term impact of vocabulary learning tasks designed based on the ILH’s work on young L2 learners at this time remains unclear. It would be interesting to study word retention in both pre-primary and primary pupils after completing the same type of task a few weeks apart, and to investigate possible causes of word retention or word loss. 

Third, future research should focus on qualitative exploration of the trajectory of performing tasks with peers and teachers in real L2 classroom practice. Therefore, classroom-based case studies of learners and teachers are needed to find out not only what tasks can and cannot benefit these age groups, but also why, and to reveal how learner learning and teacher teaching can be predicted by the ILH. 

Fourth, how measurements are selected to match the focus of the task is another area that needs further research. A systematic review of the variety of measurement tools that teachers and researchers can choose from in the literature can provide valuable insights into finding tests that are best suited to a particular design task and may reveal areas in which learners can benefit from age-appropriate measurements. In addition, more consideration and research into the effects of vocabulary testing are needed. 

### 6.2. Limitations

The current systematic review has some aspects that limit its generality, but also provide a focus for future research. For example, the databases that we utilized include only the top five databases in language and education. Further studies on a larger scale are needed to make the results more comprehensive. In addition, this review is limited to analyzing studies published in English. Therefore, further investigation is needed to examine more studies written in languages other than English. Furthermore, with our results indicating that 92% of the studies focused on ESL/EFL and only 8% on languages other than English (*k* = 6), our data may not truly represent the use of the ILH for all languages. To investigate this issue, we recalculated the results for the 22 task types after removing the studies on languages other than English (see Table S6 and Figure S2–S7 in Supplementary Materials) but found the same pattern in the support for the ILH hypothesis for all task types, except a slight difference for translation due to the removal of one of the seven studies that used translation as a vocabulary learning task. We call on more researchers to apply the ILH in languages other than English in future empirical studies. Moreover, empirical studies published after we completed our search of electronic databases (i.e., 2021) were not included in the analysis. In summary, although we initially found a large number of studies from database searches, there were inherent selection biases that could not be avoided.

## 7. Conclusions

Although the ILH was proposed more than 20 years ago and many empirical studies have been conducted on L2 vocabulary acquisition, there is still no consensus as to the predictive ability of the ILH. In this paper, 78 studies were analyzed in depth from several new angles. According to our analysis, the predictive power of the ILH for L2 vocabulary learning may be higher than previous review studies determined. Similar to Yanagisawa and Webb’s meta-analysis [8], we synthesized the tasks that strictly controlled IL according to the original ILH. Previously unexplained variance in vocabulary learning gains were explained. Our findings provided some potential task design improvements as implications for future researchers. With these findings, the current systematic review makes a step towards validating the ILH to better cater to various learners’ vocabulary learning needs.

## Figures and Tables

**Table 1:** Journals with three or more studies published.

Journal	Studies	Category	Quartile
Language Teaching Research	8	Linguistics Education and educational research	1 1
System	4	Linguistics Education and educational research	1 1
IRAL	4	Linguistics Education and educational research	2 1

**Table 2:** Prevalence of task types.

Task Type	Tasks	
	*n*	%
Complex task	72	27.48
Sentence-writing	28	10.69
Fill-in-the-blanks	28	10.69
Multiple-choice	24	9.16
Reading	24	9.16
Comprehension questions	16	6.11
Composition-writing	15	5.73
Translation	12	4.58
True/false	9	3.44
Meaning-inferring	7	2.67
Meaning-matching	5	1.91
Segments-combining	5	1.91
Short-response	3	1.15
Summarizing	3	1.15
Sentence-copying	2	0.76
Regular course	2	0.76
Open discussion	1	0.38
Form-meaning-fit	1	0.38
Sentence-rewording	1	0.38
Vocabulary Self-Collection Strategy Plus	1	0.38
Only reading the glosses	1	0.38
Making prediction	1	0.38
N.A.	1	0.38
Total	262	100.00

Note: The task types are given in descending order.

## Data Availability

All of the data used for the research are provided through the Supplementary Materials and any additional data can be provided by contacting the first author.

## Supplementary Materials

The following supporting information can be downloaded at: https://www.mdpi.com/article/10.3390/bs12100354/s1. Table S1: Specific search strings of the databases; Table S2: Coding framework for systematic review of the ILH; Table S3: The summary of 78 reviewed studies: basic information; Table S4: The summary of 78 reviewed studies: the tasks; Table S5: Specific scales for rating studies; Table S6. Prevalence of task types (language other than English as the target language); Figure S1: Prisma 2020 flow diagram; Figure S2: High frequency task types and the ILH support in English as the target language studies; Figure S3: Medium frequency task types and the ILH support in English as the target language studies; Figure S4: Low frequency task types and the ILH support in English as the target language studies; Figure S5: High frequency task types and the ILH support in studies (any language as the target language); Figure S6: Medium frequency task types and the ILH support in studies (any language as the target language); Figure S7: Low frequency task types and the ILH support in studies (any language as the target language).
