# Supplementary material for: Empirical Support for the Involvement Load Hypothesis (ILH): A Systematic Review
Source: Behav Sci (Basel). 2022 Sep 23;12(10):354. doi: 10.3390/bs12100354 (PMC9598591; doi:10.3390/bs12100354)
Supplement: Supplementary file 1 [file behavsci-12-00354-s001.zip › behavsci-1855414-supplementary.pdf]

Supplementary Table S1. Specific search strings of the databases

**ProQuest Dissertations & Theses Global (ProQuest)**
((ab("ILH") OR ti("ILH") OR diskw("ILH")) OR (ab("involvement load") OR ti("involvement load") OR diskw("involvement load")) OR (ab("task effectiveness") OR ti("task effectiveness") OR diskw("task effectiveness")) OR (ab(task-induced) OR ti(task-induced) OR diskw(task-induced)))
**AND**<br>((ab(word*) OR ti(word*) OR diskw(word*)) OR (ab(vocab*) OR ti(vocab*) OR diskw(vocab*)) OR (ab(collocation*) OR ti(collocation*) OR diskw(collocation*)) OR (ab("n gram") OR ti("n gram") OR diskw("n gram")) OR (ab(idiom*) OR ti(idiom*) OR diskw(idiom*)) OR (ab(lex*) OR ti(lex*) OR diskw(lex*)) OR (ab(chunk*) OR ti(chunk*) OR diskw(chunk*)) OR (ab(phras*) OR ti(phras*) OR diskw(phras*)) OR (ab(pattern*) OR ti(pattern*) OR diskw(pattern*)) OR (ab(formulaic*) OR ti(formulaic*) OR diskw(formulaic*)) OR (ab(figurative*) OR ti(figurative*) OR diskw(figurative*)) OR (ab(fixed-frame*) OR ti(fixed-frame*) OR diskw(fixed-frame*)) OR (ab(binomial*) OR ti(binomial*) OR diskw(binomial*)))
**LIMITS APPLIED**<br>1. Search by document title, abstract, and index terms<br>2. Unselect full-text<br>3. Select all dates<br>4. Select English only
**Educational Resources Information Center (ERIC)**

(TI "ILH" OR AB "ILH") OR (TI "involvement load" OR AB "involvement load") OR ("task effectiveness" OR AB "task effectiveness") OR (task-induced OR AB task-induced)
**AND**<br>(TI word* OR AB word*) OR (TI vocab* OR AB vocab*) OR (TI collocation* OR AB collocation*) OR (TI “n gram” OR AB “n gram”) OR (TI idiom* OR AB idiom*) OR (TI lex* OR AB lex*) OR (TI chunk* OR AB chunk*) OR (TI phras* OR AB phras*) OR (TI pattern* OR AB pattern*) OR (TI formulaic* OR AB formulaic*) OR (TI figurative* OR AB figurative*) OR (TI fixed-frame* OR AB fixed-frame*) OR (TI binomial* OR AB binomial*)
**LIMITS APPLIED**<br>1. Must be ERIC only<br>2. Select Peer Reviewed<br>3. Unselect the "full-text" option<br>4. Select all publication types<br>5. Select all intended audience<br>6. Select equivalent subjects<br>7. Unselect apply related words<br>8. Leave date of publication blank (all years-unspecified)<br>9. Select title or abstract<br>10. Select English only
**Institute of Electrical and Electronics Engineers (IEEE)**

("Document Title": "ILH" OR "Document Title": "involvement load" OR "Document Title": "task effectiveness" OR "Document Title": "task induced") OR ("Abstract": "ILH" OR "Abstract": "involvement load" OR "Abstract": "task effectiveness" OR "Abstract": "task induced") OR ("Index Terms": "ILH" OR "Index Terms": "involvement load" OR "Index Terms": "task effectiveness" OR "Index Terms": "task induced")
**AND**<br>According to the regulations of IEEE database, the terms which related to vocabulary were manually checked by the researchers.
**LIMITS APPLIED**<br>IEEE regulations:<br>1. Limit the total number of wildcards to 7<br>2. Limit search terms to 20<br>3. Limited combined search terms to 50<br>General limits:<br>4. Search by abstract, index terms, document title<br>5. Select all dates (1884-2022)<br>6. Select English only
**Web of Science (WOS)**
“ILH” (Topic) or “involvement load” (Topic) or “task effectiveness” (Topic) or task-induced (Topic)
**AND**

word* (Topic) or vocab* (Topic) or collocation* (Topic) or "n gram" (Topic) or idiom* (Topic) or lex* (Topic) or chunk* (Topic) or phras* (Topic) or pattern* (Topic) or formulaic* (Topic) or figurative* (Topic) or fixed-frame* (Topic) or binomial* (Topic)
**LIMITS APPLIED**<br>1. Choose WOS core collection<br>2. Search by topic<br>3. Select English only<br>4. Select all years (1980 – 2021)
**Scopus**
((TITLE-ABS-KEY ({ILH})) OR (TITLE-ABS-KEY ({involvement load})) OR (TITLE-ABS-KEY ({task effectiveness})) OR (TITLE-ABS-KEY ({task induced})))
**AND**<br>((TITLE-ABS-KEY (word*)) OR (TITLE-ABS-KEY (vocab*)) OR (TITLE-ABS-KEY (collocation*)) OR (TITLE-ABS-KEY ({n gram})) OR (TITLE-ABS-KEY (idiom*)) OR (TITLE-ABS-KEY (lex*)) OR (TITLE-ABS-KEY (chunk*)) OR (TITLE-ABS-KEY (phras*)) OR (TITLE-ABS-KEY (pattern*)) OR (TITLE-ABS-KEY (formulaic*)) OR (TITLE-ABS-KEY (figurative*)) OR (TITLE-ABS-KEY (fixed AND frame*)) OR (TITLE-ABS-KEY (binomial*)))
**LIMITS APPLIED**<br>1. Search by abstract, article title, keywords

2. Select all dates (1788-2021)
3. Select English only

Note. The Scopus, WOS, and ProQuest database searches were conducted on 31 July 2021. The IEEE and ERIC database searches were conducted on August 1, 2021. To improve the reliability of database retrieval, each database was retrieved twice. A comparison of the two rounds of database searches showed that the same number of articles were identified.

Supplementary Figure S1. Prisma 2020 flow diagram

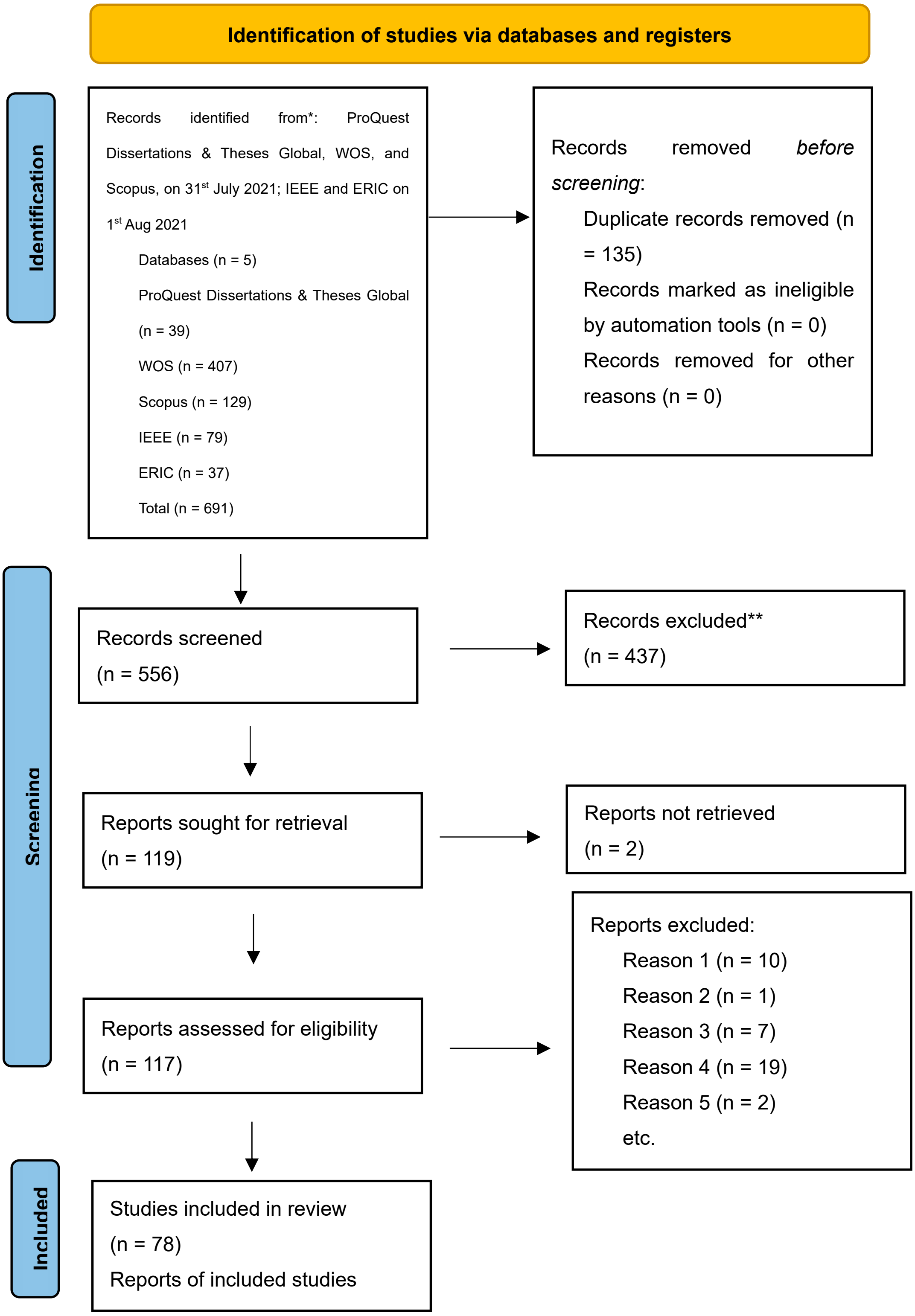

Page, M. J., McKenzie, J. E., Bossuyt, P. M., Boutron, I., Hoffmann, T. C., Mulrow, C. D., ... & Moher, D. (2021). The PRISMA 2020 statement: An updated guideline for reporting systematic reviews. *Systematic reviews*, 10(1), 1-11. https://doi.org/10.1186/s13643-021-01626-4

Supplementary Table S2. Coding framework for systematic review of the ILH

Code	Coding criteria
Publication year	When was the study published?
Author	Who wrote the study?
Country or Region	In which country or region was the study conducted?
Publication type	What publication type is this study?
Research aim/objective	What is the research aim/objective of the study?
Hypotheses	"Hypotheses are declarative statements in quantitative research in which the investigator makes a prediction or a conjecture about the outcomes of a relationship" (Creswell & Guetterman, 2019, p.623).
Theoretical framework	"A theoretical framework advances an abstract and formalized set of assumptions to guide the design and conduct of the research" (Creswell & Creswell, 2018, p.309).
Quantitative research (QUAN)	Quantitative research "is a means for testing objective theories by examining the relationship among variables" (Creswell & Creswell, 2018, p.334).
Qualitative research (QUAL)	Qualitative research "is a means for exploring and understanding the meaning individuals or groups ascribe to a social or human problem" (Creswell & Creswell, 2018, p.333).
Mixed methods research (MIXED)	Mixed methods research "is an approach to inquiry that combines or integrates both qualitative and quantitative forms of research" (Creswell & Creswell, 2018, p.331).
Research design	Research designs are "procedures for co11ecting, analyzing, and reporting research in quantitative and qualitative research (e.g., experiments)" (Creswell & Guetterman, 2019, p.628).
Target word	What words were chosen as the target words in this study?
Task type	What types of tasks were investigated in the study?
Time on task	In the study, how long did it take participants to complete the task?
Vocabulary outcome measures	What measurement tools did the study use to measure the effects of vocabulary learning tasks?
Standardized test	"A test that has been screened for reliability and validity on a large population, and calibrated on the group of test takers for whom it is intended" (García, 2003, p.432)
Researcher designed test	The researchers developed their own test and tested it on a group of participants in a specific study.
Receptive form (RF)	"What does the word sound like" (Nation, 2013, p.49)?<br>"What does the word look like" (Nation, 2013, p.49)?<br>"What parts are recognizable in this word" (Nation, 2013, p.49)?
Receptive meaning (RM)	"What parts are recognizable in this word" (Nation, 2013, p.49)?<br>"What is included in the concept" (Nation, 2013, p.49)?<br>"What others words does this word make us think of" (Nation, 2013, p.49)?

Receptive using (RU)	"In what patterns does the word occur" (Nation, 2013, p.49)?<br>"What words or types of word occur with this one "(Nation, 2013, p.49)?<br>"Where, when, and how often would we meet this word" (Nation, 2013, p.49)?
Productive form (PF)	"How is the word pronounced" (Nation, 2013, p.49)?<br>"How is the word written and spelled" (Nation, 2013, p.49)?<br>"What words parts are needed to express meaning" (Nation, 2013, p.49)?
Productive meaning (PM)	"What word form can be used to express this meaning" (Nation, 2013, p.49)?<br>"What items can be concept refer to" (Nation, 2013, p.49)?<br>"What other words could we use instead of this one" (Nation, 2013, p.49)?
Productive use (PU)	"In what patterns must we use this word" (Nation, 2013, p.49)?<br>"What words or types of words must we use with this one" (Nation, 2013, p.49)?<br>"Where, when and how often can we use this word" (Nation, 2013, p.49)?
Foreign language	Is this study about foreign language learning? What country's language?<br>E.g., A foreign language is "a language that one learns in a classroom which is situated in an unnatural linguistic setting" (Du, 2008, p.10).
Foreign language learning context	It is defined as "the circumstance where the second language learners have little exposure to the L2 outside of the classroom" (Wei, 2019, p.7).
Second language	Is this study about second language learning? What country's language?<br>E.g., A second language is a "language other than one's first language that one studies or acquires in a naturalistic setting" (Du, 2008, pp.10-11)
Second language learning context	It is defined as "the context where English is the official language and as such, it is widely used inside and outside the classroom" (Al Khalidi, 2016, p.18).
First language	What are the participants' first languages (i.e., the language that the participants learn to speak first as a child)?
Sample size	What was the actual sample size in the study?
Education level	What was the educational level of the participants?
Language proficiency level	What was the level of language proficiency of the study participants?
Learning setting	What was the learning setting?
Vocabulary learning tasks	What were the learning tasks involved in the study?

Quantitative research results	What were the main results? Were the effect sizes reported?
Qualitative research findings	What were the main findings?
Need	"Need is moderate when it is imposed by an external agent" (Laufer & Hulstijn, 2001, p.14).<br>"Need is strong when imposed on the learner by him- or herself" (Laufer & Hulstijn, 2001, p.14).
Search	"Search is the attempt to find the meaning of an unknown L2 word or trying to find the L2 word form expressing a concept by consulting a dictionary or another authority" (Laufer & Hulstijn, 2001, p.14).
Evaluation	Evaluation is moderate "when it entails recognizing differences between words, or differences between several senses of a word in a given context" (Laufer & Hulstijn, 2001, p.15).<br>Evaluation is strong when it "requires making a decision about additional words which will combine with the new word in an original sentence or text" (Laufer & Hulstijn, 2001, p.15).
Involvement Load (IL)	The IL refers to "The combination of the presence or absence of the involvement factors Need, Search, and Evaluation" (Laufer & Hulstijn, 2001, p.15).

References:

Al Khalidi, I. J. A. S. (2016). Investigating the process of EAP course design by teachers at a tertiary level, English department, a private college in Oman from the perspectives of teachers and students [EdD in TESOL, University of Exeter].

Creswell, J., & Creswell, J. D. (2018). Research Design: Qualitative, Quantitative, and Mixed Methods Approaches (5 ed.). SAGE Publications.

Creswell, J. W., & Guetterman, T. C. (2019). Educational research: Planning, conducting, and evaluating quantitative (6 ed.). Pearson.

Du, W.-H. (2008). Integrating culture learning into foreign language curricula: An examination of the ethnographic interview approach in a Chinese as a foreign language classroom The University of Wisconsin-Milwaukee].

García, P. (2003). The use of high school exit examinations in four southwestern states. *Bilingual Research Journal*, 27(3), 431-450.

Laufer, B., & Hulstijn, J. (2001). Incidental vocabulary acquisition in a second language: The construct of task-induced involvement. *Applied Linguistics*, 22(1), 1-26. https://doi.org/10.1093/applin/22.1.1

Nation, I. (2013). Learning vocabulary in another language (Vol. 10). Cambridge university press Cambridge.

Wei, L. (2019). Promoting English language proficiency through Quality Talk: An intervention with Mandarin-speaking students The Pennsylvania State University.

Supplementary Table S3. The summary of 78 reviewed studies: basic information

No.	Study<br>Author (year)	Country or region	Research approaches	N	L1	L2	Language context	Educational level
1	Hulstijn and Laufer (2001)	Israel<br>Netherlands	QUAN	186	Dutch<br>Hebrew	English	Foreign language	Tertiary
2	Hill and Laufer (2003)	Hong Kong	QUAN	96	Chinese	English	Foreign language	Tertiary
3	Beal (2007)	Canada	MIXED	118	French	English	Second language	Tertiary
4	Peters (2007)	Belgium	MIXED	84	Dutch	German	Foreign language	Tertiary
5	Keating (2008)	United States	QUAN	79	English	Spanish	Foreign language	Tertiary
6	Kim (2008)	United States	QUAN	104	Mixed	English	Second language	Mixed
7	Laufer and Girsai (2008)	Israel	QUAN	75	Hebrew	English	Foreign language	Secondary
8	Pulido (2009)	United States	QUAN	35	English	Spanish	Foreign language	Tertiary
9	Xu (2009)	China	MIXED	152	Chinese	English	Foreign language	Tertiary
10	Cheng (2011)	Taiwan	QUAN	111	Chinese	English	Foreign language	Tertiary
11	Rukholm (2011)	Canada	QUAN	66	MIXED	Italian	Foreign language	Tertiary
12	Eckerth and Tavakoli	UK	QUAN	30	MIXED	English	Second	Tertiary

	(2012)						language	
13	Maleki (2012)	Iran	QUAN	80	Hebrew	English	Foreign language	Tertiary
14	Nassaji and Hu (2012)	Canada	QUAN	32	Chinese	English	Second language	Tertiary
15	Peters (2012)	Belgium	QUAN	56	Dutch	English	Foreign language	Tertiary
16	Tsubaki (2012)	Japan	MIXED	301	Janpanese	English	Foreign language	Tertiary
17	Vosoughi (2012)	Iran	QUAN	73	Persian	English	Foreign language	Tertiary
18	Arseven (2013)	Egypt	QUAN	57	Arabic	English	Mixed	Tertiary
19	Cao (2013)	China	QUAN	70	Chinese	English	Foreign language	Tertiary
20	Touti (2013)	Iran	QUAN	64	Persian	English	Foreign language	Extra-curricular language education
21	Mármol and Sánchez-Lafuente (2013)	Spain	QUAN	28	Spanish	English	Foreign language	Primary
22	Rouhi and Mohebbi (2013)	Iran	QUAN	62	Azari-Turkish & Persian (bilingual)	English	Foreign language	Extra-curricular language education
23	Tajeddin and Daraee (2013)	Iran	QUAN	45	Persian	English	Foreign language	Extra-curricular language education
24	Jahangiri and Abilipour (2014)	Iran	QUAN	27	Persian	English	Foreign language	Extra-curricular language education
25	Li (2014)	China	MIXED	81	Chinese	English	Foreign language	Tertiary

26	Sarbazi (2014)	Iran	QUAN	30	Persian	English	Foreign language	Extra-curricular language education
27	Bao (2015)	China	QUAN	153	Chinese	English	Foreign language	Tertiary
28	Pourakbari and Biria (2015)	Iran	QUAN	150	Persian	English	Foreign language	Tertiary
29	Soleimani and Rahmanian (2015)	Iran	QUAN	33	N.A.	English	Foreign language	Extra-curricular language education
30	Wang (2015)	Taiwan	QUAN	64	Chinese	English	Foreign language	Tertiary
31	Ansarin and Bayazidi (2016)	Iran	QUAN	72	MIXED	English	Foreign language	Tertiary
32	Hu and Nassaji (2016)	Taiwan	QUAN	96	Chinese	English	Foreign language	Tertiary
33	Tang and Treffers-Daller (2016)	China	QUAN	185	Chinese	English	Foreign language	Secondary
34	Zou (2016)	China	MIXED	104	Chinese	English	Foreign language	Tertiary
35	Alharbi (2017)	Saudi Arabia	QUAN	129	Arabic	English	Foreign language	Tertiary
36	Dowswell (2017)	United Arab Emirates	QUAN	29	Arabic	English	Foreign language	Tertiary
37	Lee and Pulido (2017)	South Korea	QUAN	135	Korean	English	Foreign language	Secondary
38	Teng (2017)	China	QUAN	90	Chinese	English	Foreign language	Tertiary
39	Qin and Teng (2017)	China	MIXED	60	Chinese	English	Foreign language	Tertiary

40	Rassaei (2017)	Iran	QUAN	88	Farsi	English	Foreign language	Extra-curricular language education
41	Sauer (2016)	Denmark	MIXED	269	MIXED	English	Foreign language	Secondary
42	Snoder (2017)	Sweden	QUAN	59	Swedish	English	Foreign language	Secondary
43	Tahmasbi and Farvardin (2017)	Iran	QUAN	130	Persian	English	Foreign language	Secondary
44	F. Teng (2017)	China	QUAN	77	Chinese	English	Foreign language	Tertiary
45	Tong (2017)	China	QUAN	96	Chinese	English	Foreign language	Tertiary
46	Yang et al. (2017)	China	QUAN	81	Chinese	English	Foreign language	Tertiary
47	Zou (2017)	China	MIXED	147	Chinese	English	Foreign language	Tertiary
48	Alkhudiry (2018)	England	QUAN	40	Arabic	English	Second language	Tertiary
49	Gohar et al. (2018)	Iran	QUAN	90	Persian	English	Foreign language	Extra-curricular language education
50	Huang (2018)	China	MIXED	N.A.	Chinese	English	Foreign language	Tertiary
51	Ong and Zhang (2018)	Singapore	QUAN	154	Chinese	English	Second language	Tertiary
52	Silva and Otwinowska (2018)	Poland	QUAN	38	Polish	English	Foreign language	Primary
53	Alanazi (2019)	USA	MIXED	4	Arabic	English	Second language	Tertiary

54	Alavinia and Rahimi (2019)	Iran	QUAN	125	Persian	English	Foreign language	Tertiary
55	Bao (2019)	China	QUAN	167	Chinese	English	Foreign language	Tertiary
56	Dai et al. (2019)	China	QUAN	70	Chinese	English	Foreign language	Tertiary
57	Kıvrak (2019)	Turkey	QUAN	236	Turkish	English	Foreign language	Tertiary
58	Nguyen and Boers (2019)	Vietnam	QUAN	64	Vietnamese	English	Foreign language	Tertiary
59	Alahmadi and Foltz (2020)	Saudi Arabia	QUAN	61	Arabic	English	Foreign language	Tertiary
60	Alarjani (2020)	Saudi Arabia	QUAN	30	Arabic	English	Foreign language	Tertiary
61	Arabiana et al. (2020)	Philippines	MIXED	6	Cebuano	English	Foreign language	Primary
62	Chiu and Chen (2020)	Taiwan	QUAN	32	Chinese	English	Foreign language	Primary
63	Danilina and Shabunina (2020)	Ukraine	MIXED	19	Ukrainian	English	Foreign language	Tertiary
64	Dongho (2020)	Korea	QUAN	29	Korean	English	Foreign language	Tertiary
65	Kaivanpanah et al. (2020)	Iran	QUAN	120	N.A.	English	Foreign language	Extra-curricular language education
66	Kamali et al. (2020)	Iran	QUAN	66	Farsi	English	Foreign language	Extra-curricular language education
67	Namaziandost et al. (2020)	Iran	QUAN	40	Persian	English	Foreign language	Tertiary

68	Rassaei (2020)	Iran	QUAN	47	Persian	English	Foreign language	Extra-curricular language education
69	Xu and Zhang (2020)	USA	MIXED	22	English	Chinese	Foreign language	Tertiary
70	Yang and Cao (2020)	China	QUAN	144	Chinese	English	Foreign language	Extra-curricular language education
71	Alcaraz-Marmol (2021)	Spain	QUAN	60	Spanish	English	Foreign language	Primary
72	Ansarin and Khabbazi (2021)	Iran	QUAN	204	Persian	English	Foreign language	Tertiary
73	Danilina (2021)	Ukraine	MIXED	29	Ukrainian	English	Foreign language	Tertiary
74	Hanks (2021)	Mixed	QUAN	19	MIXED	English	Foreign language	Extra-curricular language education
75	San Mateo-Valdehita and de Diego (2021)	France	QUAN	308	French	Spanish	Foreign language	Tertiary
76	Silva et al. (2021)	Poland	QUAN	39	Polish or Slavic languages	English	Foreign language	Tertiary
77	Taheri and Golandouz (2021)	Iran	QUAN	78	Persian	English	Foreign language	Extra-curricular language education
78	Teng and Zhang (2021)	China	QUAN	120	Chinese	English	Foreign language	Tertiary

Supplementary Table S4. The summary of 78 reviewed studies: the tasks

No.	Study<br>Author (year)	Task	The IL was provided by the original author.	The IL was re-calculated in this review.
1	Hulstijn and Laufer (2001)	1. Reading + glosses (1+0+0)<br>2. Reading + glosses + fill-in-the blanks (1+0+1)<br>3. Composition-writing + glosses (1+0+2)	Yes	No
2	Hill and Laufer (2003)	1. Reading + E-dictionary + yes/no questions (1+1+1)<br>2. Reading + E-dictionary + multiple-choice (1+1+1)<br>3. Reading + E-dictionary + multiple-choice (1+1+1)<br>4. Control: Reading (0+0+0)	No	Yes
3	Beal (2007)	1. Reading + glosses +highlighting (1+0+1)<br>2. Reading + multiple-choice (1+1+1)<br>3. Reading +dictionary + sentences-writing (1+2+2)<br>4. Control: Reading (0+0+0)	Yes	Yes
4	Peters (2007)	1. Reading + E-dictionary + comprehension questions with plus relevant words and minus relevant words in incidental learning context (1+1+0)<br>2. Reading + E-dictionary + comprehension questions with plus relevant words and minus relevant words in intentional learning context (1+1+0)	No	Yes
5	Keating (2008)	1. Reading + glosses + true/false (1+0+0)<br>2. Reading + glosses (8 target words + 4 distractors) on a separate page + fill-in-the-blanks + true/false (1+0+1)<br>3. Glosses (8 target words + 4 distractors) + sentences-writing (using the target words) (1+0+2)	Yes	No
6	Kim (2008)	Experiment 1:	Yes	No

		1. Reading + glosses + comprehension questions (1+0+0)<br>2. Reading + glosses (target words + 5 distractors) on a separate page + comprehension questions + fill-in-the-blanks (1+0+1);<br>3. Composition-writing + glosses (1+0+2)<br>Experiment 2:<br>1. Composition-writing + glosses (1+0+2)<br>2. Sentences-writing + glosses (1+0+2)		
7	Laufer and Girsai (2008)	Meaning focused instruction (MFI):<br>1. Reading + comprehension questions (0+0+0) or (1+1+1)<br>2. Reading + open discussion （0+0+0） or (1+1+1)<br>Note: During these activities, some of the target words may have been used by some learners. However, they were not singled out for teaching.<br>Form focused instruction (FFI):<br>1. Reading + multiple-choice (1+1+0)<br>2. Reading + fill-in-the-blanks (1+1+1)<br>Contrastive analysis and translation (CAT)<br>1. Reading + translation (from L2 to L1) (1+1+1)<br>2. Reading + translation (from L1 to L2) (1+1+2)	Yes	No
8	Pulido (2009)	1. Reading + translation (familiar topic) (1+1+0)<br>2. Reading + translation (unfamiliar topic) (1+0+1)	No	Yes
9	Xu (2009)	1. Reading + glosses + multiple-choice (1+0+0)<br>2. Reading + glosses + fill-in-the-blanks (1+0+1)<br>3. Reading + glosses + sentences-writing (1+0+2)	No	Yes
10	Cheng (2011)	1. Reading with target words highlighted + glosses on a separate page (1+0+0)<br>2. Reading + fill-in-the-blank + word list on a separate page + E-	Yes	No

		dictionary (1+1+1)<br>3. Reading + word list on a separate page + E-dictionary +sentences-writing (1+1+2)		
11	Rukholm (2011)	High IL tasks<br>Task set 1:<br>1. Reading + discussion with peers + meaning-inferring (2+1+1)<br>2. Dictionary + sentences-writing (1+1+2)<br>Task set 2:<br>1. Fill-in-the-blanks + word list (12 target words + 2 distractors) (1+1+1)<br>Task set 3:<br>1. Composition-writing + word list (5 target words) +meaning-inferring (2+1+2)<br>Low IL tasks<br>1. Reading + glosses + irrelevant comprehension questions (0+0+0)<br>2. Reading + glosses + irrelevant true/false questions (0+0+0)<br>3. Reading + glosses + comprehension questions (L1) + response in L1 sentences (1+0+0)<br>4. Glosses + irrelevant fill-in-the-blanks (0+0+0)<br>5. Glosses + comprehension questions (L1 mixed with L2) + response in L1 sentences (1+0+0)	Yes	No
12	Eckerth and Tavakoli (2012)	1. Reading + marginal glosses (1+0+0)<br>2. Fill-in-the-blanks + word list (target words + definitions) (1+0+1)<br>3. Reading +marginal glosses + composition-writing (1+0+2)	Yes	No
13	Maleki (2012)	1. Listening + irrelevant multiple-choice + glosses（0+0+0）	Yes	No

		2. Listening + multiple-choice + glosses (1+0+0)<br>3. Listening + multiple-choice + glosses + sentences-writing (1+0+2)		
14	Nassaji and Hu (2012)	1. Reading + marginal multiple-choice glosses (1+0+1)<br>2. Reading + meaning-inferring (1+1+1)<br>3. Reading (i.e., the derivational text) + form-meaning-fit (1+2+2)	Yes	No
15	Peters (2012)	Message-oriented group:<br>1. Reading + marginal glosses +relevant comprehension questions in L1 (1+0+0)<br>Vocabulary-oriented group:<br>2. Reading + marginal glosses + translation + multiple-choice (1+0+1)	No	Yes
16	Tsubaki (2012)	1. Multiple-choice + sentences-writing in L1 (1+1+2)<br>2. Multiple-choice + sentences-copying in L2 (1+1+1)	Yes	Yes
17	Vosoughi (2012)	1. Reading + reading (i.e., other passages with the same target words) + irrelevant comprehension questions (1+1+0)<br>2. Reading + dictionary work (i.e., copy example sentence, synonym, and pronunciation from dictionary) (1+1+0)<br>3. Reading + translation (1+1+0)	Yes	Yes
18	Arseven (2013)	1. Reading + multiple-choice + E-dictionary (1+1+0)<br>2. Reading + multiple-choice + glosses (1+0+0)	No	Yes
19	Cao (2013)	1. Reading + comprehension questions + glosses at the bottom (1+0+0)<br>2. Reading + reading comprehension questions + glosses (10 target words + 5 distractors) on a separate page + fill-in-the-blanks (1+0+1)<br>3. Reading + reading comprehension questions +glosses at the	Yes	No

		bottom + sentences-writing (1+0+2)		
20	Touti (2013)	1. Reading + fill-in-the-blanks + glosses on a separate page + comprehension questions (1+0+1)<br>2. Reading + glosses on a separate page + composition-writing (1+0+2)	No	Yes
21	Mármol and Sánchez-Lafuente (2013)	1. Reading + comprehension questions + glosses (1+0+0)<br>2. Reading + comprehension questions + fill-in-the-blanks + glosses (18 target words + 9 distractors) (1+0+1)<br>3. Glosses + sentences-writing (1+0+2)<br>4. Dictionary + sentences-writing (1+1+2)	Yes	No
22	Rouhi and Mohebbi (2013)	1. Reading + E-glosses (i.e., pictorial & sound features) (1+0+0)<br>2. Reading + E-glosses (i.e., pictorial features) (1+0+0)<br>3. Reading + E-glosses (i.e., video features) (1+0+0)<br>4. Control: Reading + teacher instructions in regular classes (1+0+0)	No	Yes
23	Tajeddin and Daraee (2013)	1. Control: Reading + irrelevant comprehension questions (0+0+0)<br>2. Message-oriented task: Reading + true/false (1+1+0)<br>3. Form-oriented task: Reading + meaning-matching (1+1+1)	Yes	No
24	Jahangiri and Abilipour (2014)	Individual<br>1. Sentences-writing + dictionary (i.e., 3 target words) (1+0+2)<br>2. Fill-in-the-blanks + dictionary (i.e., 2 target words + 1 distractor) (1+0+1)<br>Collaborative<br>3. Sentences-writing + dictionary (i.e., 3 target words) (1+0+2)<br>4. Fill-in-the-blanks + dictionary (i.e., 2 target words + 1 distractor) (1+0+1)	Yes	Yes
25	Li (2014)	1. Reading + irrelevant true/false questions (0+0+0)	Yes	No

		2. Reading + relevant true/false questions (1+1+0)<br>3. Reading + fill-in-the-blanks (i.e., 9 target words + 5 distractors) + relevant true/false questions (1+1+1)<br>4. Reading + sentences-writing (1+1+2)		
26	Sarbazi (2014)	1. Reading + glosses + irrelevant true/false (0+0+0)<br>2. Reading + glosses + true/false (1+0+0)<br>3. Reading + glosses + true/false + composition-writing (1+0+2)	Yes	Yes
27	Bao (2015)	1. Control: Reading + glosses + irrelevant matching (0+0+0)<br>2. Reading + glosses + translation (1+0+0)<br>3. Reading + glosses + fill-in-the-blanks (1+0+1)<br>4. Reading + glosses + segments-combining (1+0+1)<br>5. Reading + glosses + sentences-writing (1+0+2)	Yes	No
28	Pourakbari and Biria (2015)	Receptive vocabulary tasks:<br>1. Reading + glosses + graphic organizers + true/false (1+0+0)<br>2. Reading + glosses + graphic organizers + matching (1+0+1)<br>3. Reading + dictionary + multiple-choice (1+1+1)<br>Productive vocabulary tasks:<br>1. Reading + glosses + short-response (1+0+0)<br>2. Reading + glosses + graphic organizers + fill-in-the-blanks (1+0+1)<br>3. Reading + glosses + sentence-writing (1+0+2)	Yes	No
29	Soleimani and Rahmanian (2015)	1. Reading + glosses in L2 + fill-in-the-blanks + irrelevant comprehension questions (1+0+1)<br>2. Reading + glosses in L1 + irrelevant comprehension questions + sentences-writing (1+0+2)<br>3. Reading + glosses in L1 + multiple-choice (1+0+0)	Yes	No
30	Wang (2015)	1. Reading (0+0+0)<br>2. Reading + glosses (1+0+0)	Yes	Yes

		3. Reading + glosses + fill-in-the-blanks (1+0+1)<br>4. Reading + glosses + fill-in-the-blanks + sentences-writing (1+0+2)<br>Note: The author calculated the SUM of different tasks as accumulated loads.<br>Task 1 + Task 2 = 1<br>Task 1 + Task 2 + Task 3 = 3<br>Task 1 + Task 2 + Task 3 + Task 4 = 5		
31	Ansarin and Bayazidi (2016)	Task type was used as a within-subject factor<br>1. Glosses in a mini-dictionary format (18 target words + 3 distractors) + multiple-choice * 3 sets (1+0+0)<br>2. Glosses in a mini-dictionary format (18 target words + 3 distractors) + fill-in-the-blanks * 3 sets (1+0+1)<br>3. Glosses in a mini-dictionary format (18 target words + 3 distractors) + sentences-writing (1+0+2)	Yes	Yes
32	Hu and Nassaji (2016)	1. Reading + multiple-choice (1+1+0)<br>2. Reading + multiple-choice (definition) (1+1+0)<br>3. Reading + glosses on a separate page (14 target words + extra distractors) + fill-in-the-blanks (1+0+1)<br>4. Reading + sentences-rewording (1+1+1)	Yes	Yes
33	Tang and Treffers-Daller (2016)	1. Reading + glosses in the text (0+0+0)<br>2. Reading + glosses in the text + irrelevant multiple-choice (0+0+0)<br>3. Reading + glosses in the end + multiple-choice (1+0+0)<br>4. Reading + marginal glosses with multiple meanings + multiple-choice (1+0+1)<br>5. Reading + glosses in the end with multiple meanings + multiple-choice (1+0+1)	Yes	Yes

		6. Reading + marginal glosses with multiple meanings + sentences-writing (1+0+2)		
34	Zou (2016)	1. Reading + dictionary use + multiple choice (1+1+0)<br>2. Reading + meaning-inferring + irrelevant multiple choice (1+1+0)	No	Yes
35	Alharbi (2017)	1.Teacher-fronted/non-corpus assisted CAT (contrastive analysis and translation) condition +L2 to L1 translation (1+1+1)<br>2. Teacher-fronted/ non-corpus assisted CAT condition +L1 to L2 translation (1+1+2)<br>3. Corpus-assisted CAT + L2 to L1 translation (1+2+1)<br>4. Corpus-assisted CAT + L1 to L2 translation (1+2+2)<br>5. Corpus-assisted non-CAT condition + multiple-choice (1+1+1)<br>6. Corpus-assisted non-CAT condition + fill-in-the-blanks (1+1+1)	Yes	Yes
36	Dowswell (2017)	1. Vocabulary Self-Collection Strategy Plus (VSS+) + wiki (2+2+2)<br>2. Control: regular course (0+0+0)	No	Yes
37	Lee and Pulido (2017)	Reading (passage 1) + interest rating scale + irrelevant multiple-choice in L1 + reading (passage 2) + interest rating scale + irrelevant multiple-choice in L1 + intervening number task (0+0+0)	No	Yes
38	Teng (2017)	1. Reading + glosses (1+0+0)<br>2. Reading + word list (target words + distracters) + fill-in-the-blanks (1+0+1)<br>3. Reading + glosses + composition-writing (1+0+2)	Yes	No

39	Qin and Teng (2017)	1. Reading + glosses (1+0+0)<br>2. Reading + word list (target words + distracters) + fill-in-the-blanks (1+0+1)<br>3. Reading + glosses + composition-writing (1+0+2)	Yes	No
40	Rassaei (2017)	Summarizing condition:<br>1. [Output instruction session: Reading + intervention (i.e., Teacher taught summarization rules)] + [Output activity session: Reading + comprehension questions in L1 + Task 1 (i.e., Glosses + summaries-writing)] (1+0+2)<br>Predicting condition:<br>2. [Output instruction session: Reading + intervention (i.e., Teacher taught prediction strategy)] + [Output activity session: Reading + comprehension questions in L1 + Task 2 (i.e., Glosses + predictions-writing)] (1+0+2)<br>Questioning/answering condition:<br>3. [Output instruction session: Reading + intervention (i.e., Teacher taught comprehension question generating skills)] + [Output activity session: Reading + comprehension questions in L1 + Task 3 (i.e., Glosses + comprehension questions and answers-writing)] (1+0+2)<br>Control:<br>4. [No output instruction session] + [Activity session: Reading + comprehension questions in L1 + Task 4 (i.e., True/false)] (1+0+0)	No	Yes
41	Sauer (2016)	1. Reading + bold-printing + multiple-choice & true/false & short-response + irrelevant post-reading exercises (1+0+1)<br>2. Reading + glosses + multiple-choice & true/false & short-	No	Yes

		response + irrelevant post-reading exercises (1+0+1)<br>3. Reading + bold-printing & glosses + multiple-choice & true/false & short-response + irrelevant post-reading exercises (1+0+1)<br>4. Control: Reading + multiple-choice & true/false & short-response + irrelevant post-reading exercises (1+1+1)		
42	Snoder (2017)	1. Listening + fill-in-the-blanks + glosses (1+0+1)<br>2. Listening + sentences-writing + glosses (1+0+2)<br>3. Listening + fill-in-the-blanks + L1 glosses + dictionary (1+1+1)<br>4. Listening + sentences-writing + L1 glosses + dictionary (1+1+2)	Yes	No
43	Tahmasbi and Farvardin (2017)	1. [Reading task: Reading + glosses in text] + [Task 1: Dictionary + composition-writing (1+1+2)]<br>2. [Reading task: Reading + glosses in text] + [Task 2: Dictionary + sentences-writing (1+1+2)]<br>3. [Reading task: Reading + glosses in text] + [Task 3: Segments-combining (1+0+1)]<br>4. [Reading task: Reading + glosses in text] + [Task 4: Dictionary + translation (1+1+0)<br>5. [Reading task: Reading + glosses in text] + [Task 4: Fill-in-the-blanks + word list (1+0+1)<br>6. Control: [Reading task: Reading + glosses in text] + [Task 6: Irrelevant matching (0+0+0)]	Yes	Yes
44	F. Teng (2017)	1. Reading + dictionary (1+1+0)<br>2. Reading + dictionary + fill-in-the-blanks + word list (1+1+1)<br>3. Reading + dictionary +composition-writing (1+1+2)	Yes	No
45	Tong (2017)	1. Reading + glosses + translation (1+0+0)	No	Yes

		2. Reading + glosses in multiple-choice format + translation (1+0+1)<br>3. Reading + glosses in multiple-choice format + short-response (1+0+1)<br>4. Reading + glosses + short-response (1+0+0)		
46	Yang et al. (2017)	1. Reading + glosses + irrelevant multiple-choice + sentences-writing (1+0+2)<br>2. Reading + glosses + irrelevant multiple-choice + fill-in-the-blanks + glosses (8 target words + 4 distracters) (1+0+1)<br>3. Reading + glosses + irrelevant multiple-choice + irrelevant short-response (0+0+0)<br>4. Control: Normal class (0+0+0)	Yes	Yes
47	Zou (2017)	1. Fill-in-the-blanks in a reading text + glosses (1+0+1)<br>2. Sentences-writing + glosses (1+0+2)<br>3. Composition-writing + glosses (1+0+2)	Yes	No
48	Alkhudiry (2018)	Elaboration task:<br>1. Reading + glosses +multiple-choice + sentences-writing (1+0+2)<br>2. Reading + glosses +multiple-choice + sentences-writing (distractor version that did not contain non-words) (0+0+0)	Yes	Yes
49	Gohar et al. (2018)	1. Glosses + sentences-writing (1+0+2)<br>2. Glosses + composition-writing (1+0+2)<br>3. Reading + glosses + short-response (1+0+0)	Yes	Yes
50	Huang (2018)	1. Reading + irrelevant comprehension questions + glosses + true/false (1+0+0)<br>2. Reading + irrelevant comprehension questions + dictionary (1+1+0)<br>3. Reading + irrelevant comprehension questions + glosses +	Yes	No

		segments-combination (1+0+1)<br>4. Reading + irrelevant comprehension questions + dictionary + fill-in-the-blanks (1+1+1)<br>5. Reading + irrelevant comprehension questions + glosses + sentences-writing (1+0+2)<br>6. Reading + irrelevant comprehension questions + dictionary + sentences-writing (1+1+2)		
51	Ong and Zhang (2018)	Code-switched (CS) reading task: L1 text with L2 target words<br>1. Reading + meaning-inferring (1+1+0)<br>Control: L2 text<br>2. Reading + meaning-inferring (1+1+0)	Yes	Yes
52	Silva and Otwinowska (2018)	1. Reading + glosses + sentences-writing (1+0+2)<br>2. Reading + glosses + [multiple-choice comprehension questions (L1)] + [wordlist+ matching (i.e., pictures)] (1+0+1)<br>3. Reading + glosses in multiple-choice format (L1) + [multiple-choice comprehension questions (L1)] + [creating associations (i.e., L1 or L2)] (1+1+1)	Yes	Yes
53	Alanazi (2019)	1. Reading + glosses + sentences-writing (1+0+1)<br>2. Reading + glosses + fill-in-the-blanks (i.e., 6 target words + 2 distractors) (1+0+2)	Yes	No
54	Alavinia and Rahimi (2019)	1. Reading + glosses in the text + dictionary + sentences-writing (1+0+2)<br>2. Reading + glosses in the text + definition (1+0+0)<br>3. Reading + glosses in the text + fill-in-the-blanks (1+0+1)<br>4. Reading + glosses in the text + segments combination (1+0+1)<br>5. Control: Reading + glosses + irrelevant matching (0+0+0)	Yes	Yes
55	Bao (2019)	1. Reading + glosses in the text + matching (1+0+1)<br>2. Reading + glosses in the text + fill-in-the-blanks (1+0+1)	Yes	No

		3. Reading + glosses in the text + multiple-choice (1+0+1)<br>4. Reading + glosses in the text + segments combination (1+0+1)		
56	Dai et al. (2019)	1. Reading [E-dictionary + conceptual metaphor information (i.e., salient) + 7-10 example sentences] + multiple-choice (1+1+1)<br>2. Reading [E-dictionary + conceptual metaphor information (i.e., not salient) + 1 example sentence] + multiple-choice (1+2+1)<br>3. Control: Reading [E-dictionary + 1 example sentence] + multiple-choice (1+2+1)	Yes	No
57	Kıvrak (2019)	1. Control: Brief irrelevant discussion + reading + irrelevant comprehension questions & answer check + irrelevant peer-discussions (0+0+0)<br>2. Control: Brief irrelevant discussion + watching (i.e., video podcasts) + irrelevant comprehension questions & answer check +irrelevant peer-discussions (0+0+0)<br>3. Brief irrelevant discussion + fill-in-the-blanks in a reading text + glosses + answer check + irrelevant comprehension questions & answer check +irrelevant peer-discussions (1+0+1)<br>4. Brief irrelevant discussion + watching (i.e., video podcasts) + glosses + fill-in-the-blanks in selected sentences + answer check + 2nd watching + irrelevant comprehension questions & answer check +irrelevant peer-discussions (1+0+1)<br>5. Brief irrelevant discussion + reading + glosses + target word in bold print + less irrelevant comprehension questions & answer check + sentences-writing + answer check + irrelevant peer-discussions (1+0+2)<br>6. Brief irrelevant discussion + glosses + watching (i.e., video	Yes	No

		podcasts) + 2nd watching + less irrelevant comprehension questions & answer check + sentences-writing + answer check + irrelevant peer-discussions (1+0+2)		
58	Nguyen and Boers (2019)	1. Watching (i.e., TED Talk) + note-taking + summarizing (i.e., orally) + 2nd watching + yes/no (i.e., without access to TED Talk) (1+0+2)<br>2. Watching (i.e., TED Talk) + note-taking + revise and organize note + 2nd watching + yes/no (i.e., without access to TED Talk) (1+0+1)	No	Yes
59	Alahmadi and Foltz (2020)	1. Reading + meaning-inferring (1+1+0)<br>2. Reading + dictionary (1+1+0)	No	Yes
60	Alarjani (2020)	1. Glosses + sentences-writing (1+0+2)<br>2. Control: Glosses (1+0+0)	No	Yes
61	Arabiana et al. (2020)	1. Watching +various ungraded tasks (IL=N.A.)	No	No
62	Chiu and Chen (2020)	Traditional face-to-face storytelling session:<br>1. Listening + reading (i.e., the Keynote) +watching (i.e., the scaffolds from the storyteller's face or body) + word-meaning focused comprehension questions + summarizing (i.e., peer and group discussions) (1+0+2)<br>Computer-mediated communication (CMC) storytelling session:<br>2. Watching (i.e., the picture book and animation) + listening + word-meaning focused comprehension questions + summarizing (i.e., peer and group discussions) (1+0+2)	Yes	Yes
63	Danilina and Shabunina (2020)	Intentional:<br>1. Attending vocabulary lesson (i.e., in class) + composition-writing (i.e., at home) (1+0+2)<br>Incidental:<br>2. Watching (i.e., online video at home) + irrelevant	Yes	No

		composition-writing (i.e., at home) (1+1+2)		
64	Dongho (2020)	1. Reading + target words in bold + dictionary + 5 multiple-choice comprehension questions + 5 multiple-choice vocabulary questions (1+1+0)<br>2. Fill-in-the-blanks (i.e., in text) + glosses + 5 multiple-choice comprehension questions (1+0+1)<br>3. Fill-in-the-blanks (i.e., in text) + word list + dictionary (1+1+1)	Yes	No
65	Kaivanpanah et al. (2020)	Input tasks:<br>1. Reading + dictionary + comprehension questions (1+1+1)<br>2. Reading + glosses + irrelevant comprehension questions (0+0+0)<br>Output tasks:<br>3. Reading +fill-in-the-blanks (i.e., in the text) + glosses (10 target words + 5 distractors) on a separate page + comprehension questions (1+0+1)<br>4. Reading + glosses +composition-writing (1+0+2)<br>Phase 1: Task 1 vs. Task 4<br>Phase 2: Task 1 vs. Task 3<br>Phase 3: Task 2 vs. Task 3	Yes	No
66	Kamali et al. (2020)	1. Reading + glosses (i.e., in the text) + summarizing (i.e., orally) (1+0+2)<br>2. Reading + glosses (i.e., in the text) + summarizing (i.e., writing) (1+0+2)<br>3. Control: Reading + glosses (i.e., in the text) + true/false (1+0+0)	Yes	No
67	Namaziandost et al. (2020)	1. Reading + dictionary + comprehension questions (1+1+1)<br>2. Fill-in-the-blanks in the text + glosses (1+0+1)	Yes	Yes

68	Rassaei (2020)	1. E-reading + E-dynamic glosses (1+1+1)<br>2. E-reading + glosses (1+0+0)<br>3. Control: E-reading (0+0+0)	Yes	No
69	Xu and Zhang (2020)	1. Meaning-inferring (1+1+0)<br>2. Meaning-inferring + context (i.e., sentences) (1+1+1)	No	Yes
70	Yang and Cao (2020)	1. Reading + comprehension questions + graphic organizer + irrelevant glosses (0+0+0)<br>2. Reading + comprehension questions + graphic organizer + glosses (1+0+0)<br>3. Fill-in-the-blanks (i.e., in the text) + irrelevant comprehension questions + glosses (8 target words +2 distractors) (1+0+1)<br>4. Fill-in-the-blanks (i.e., in the text) + irrelevant comprehension questions + dictionary (1+1+1)<br>5. Reading + irrelevant comprehension questions+ glosses + sentences-writing (1+0+2)	Yes	No
71	Alcaraz-Marmol (2021)	1. Reading + meaning inferring + dynamic glosses (1+1+1)<br>2. Reading + glosses (1+0+0)<br>3. Control: Reading (0+0+0)	No	Yes
72	Ansarin and Khabbazi (2021)	1. Listening + multiple-choice comprehension questions + sentences-writing + glosses (1+0+2)<br>2. Listening + multiple-choice comprehension questions + fill-in-the-blanks (i.e., in the text) + glosses (20 target words + 4 distractors) (1+0+1)<br>3. Listening + multiple-choice comprehension questions + summarizing (i.e., writing) (0+0+0)	Yes	No
73	Danilina (2021)	Incidental:<br>1. Composition-writing + reading & find collocations + peer discussion + fill-in-the-blanks (1+2+0)	Yes	Yes

		Intentional:<br>2. Reading & find collocations + peer discussion + composition-writing (1+2+2)<br>3. Translation (i.e., an artificial need) + reading & find collocations +memorizing (1+1+1)		
74	Hanks (2021)	1. Virtual, pre-recorded Zoom class session (i.e., vocabulary teaching) + multiple-choice (1+0+1)<br>2. Virtual, pre-recorded Zoom class session (i.e., vocabulary teaching) + fill-in-the-blank (1+0+1)<br>3. Virtual, pre-recorded Zoom class session (i.e., vocabulary teaching) + sentences-writing (1+0+2)	No	Yes
75	San Mateo-Valdehita and de Diego (2021)	1. Multiple-choice + glosses (1+0+0)<br>2. Fill-in-the-blanks + glosses (1+0+1)<br>3. Sentence-writing + glosses (1+0+2)	Yes	No
76	Silva et al. (2021)	1. Glosses + sentences-writing (1+0+2)<br>2. Glosses + composition-writing (1+0+2)	No	Yes
77	Taheri and Golandouz (2021)	1. Reading + glosses + sentences-writing without the original text (1+0+2)<br>2. Reading + glosses + summarizing without the original text (1+0+2)<br>3. Reading (i.e., incomplete stories) + glosses + making predictions (1+0+2)	Yes	No
78	Teng and Zhang (2021)	1. Reading + glosses (1+0+0)<br>2. Reading + fill-in-the-blanks + glosses (i.e., target words + distracters) (1+0+1)<br>3. Reading + glosses + composition writing (1+0+2)<br>4. Reading + glosses+ E-dictionary + composition writing	Yes	Yes

		(1+1+2)		

Note: The references listed below are in the same order as Table S3 and Table S4.

1. Hulstijn, I. H., & Laufer, B. (2001). Some empirical evidence for the involvement load hypothesis in vocabulary acquisition. *Language Learning*, 51(3), 539-558. https://doi.org/10.1111/0023-8333.00164
2. Hill, M., & Laufer, B. (2003). Type of task, time-on-task and electronic dictionaries in incidental vocabulary acquisition. *IRAL - International Review of Applied Linguistics in Language Teaching*, 41(2), 87-106. https://doi.org/10.1515/iral.2003.007
3. Beal, V. (2007). The weight of involvement load in college level reading and vocabulary tasks (Publication No. 85705044) [Master's thesis, Concordia University]. ProQuest Dissertations and Theses Global.
4. Peters, E. (2007). Manipulating L2 learners' online dictionary use and its effect on L2 word retention. *Language Learning & Technology*, 11(2), 36-58.
5. Keating, G. D. (2008). Task effectiveness and word learning in a second language: The involvement load hypothesis on trial. *Language Teaching Research*, 12(3), 365-386. https://doi.org/10.1177/1362168808089922
6. Kim, Y. (2008). The role of task-induced involvement and learner proficiency in L2 vocabulary acquisition. *Language Learning*, 58(2), 285-325. https://doi.org/10.1111/j.1467-9922.2008.00442x
7. Laufer, B., & Girsai, N. (2008). Form-focused instruction in second language vocabulary learning: A case for contrastive analysis and translation. *Applied Linguistics*, 29(4), 694-716. https://doi.org/10.1093/applin/amn018
8. Pulido, D. (2009). How involved are American L2 learners of Spanish in lexical input processing tasks during reading? *Studies in Second Language Acquisition*, 31(1), 31-58. https://doi.org/10.1017/S0272263109090020
9. Xu, J. (2009). An experimental study on the effects of different reading tasks on L2 vocabulary acquisition. *English Language Teaching*, 2(3), 69-79.
10. Cheng, H.-C. (2011). Vocabulary acquisition in learning English as a second language: Examining the involvement load hypothesis and language anxiety with Taiwanese college students (Publication No. 1031154652) [Doctoral dissertation, University of Northern Colorado]. ProQuest Dissertations and Theses Global.
11. Rukholm, V. N. (2011). *Facilitating lexical acquisition in beginner learners of Italian through popular song* (Publication No. 1323346428) [Doctoral dissertation, University of Toronto]. ProQuest Dissertations and Theses Global.
12. Eckerth, J., & Tavakoli, P. (2012). The effects of word exposure frequency and elaboration of word processing on incidental L2 vocabulary acquisition through reading. *Language Teaching Research*, *16*(2), 227-252. https://doi.org/10.1177/1362168811431377
13. Maleki, N. A. (2012). The effect of the involvement load hypothesis on improving Iranian EFL learners' incidental vocabulary acquisition in

listening comprehension classes. *Australian Journal of Basic and Applied Sciences*, *6*(9), 119-128.

14. Nassaji, H., & Hu, H. C. M. (2012). The relationship between task-induced involvement load and learning new words from context. *Iral-International Review of Applied Linguistics in Language Teaching*, *50*(1), 69-86. https://doi.org/10.1515/iral-2012-0003
15. Peters, E. (2012). The differential effects of two vocabulary instruction methods on EFL word learning: A study into task effectiveness. *Iral-International Review of Applied Linguistics in Language Teaching*, *5*(3), 213-238. https://doi.org/10.1515/iral-2012-0009
16. Tsubaki, M. (2012). Vocabulary learning with graphic organizers in the EFL environment: Inquiry into the involvement load hypothesis (Publication No. 1267036105) [Doctoral dissertation, Temple University]. ProQuest Dissertations and Theses Global.
17. Vosoughi, M. (2012). Enhancing lexical knowledge though L2 medium tasks. *English Language Teaching*, 5(10), 34-42. https://doi.org/10.5539/elt.v5n10p34
18. Arseven, S. (2013). The effects of marginal glosses and online dictionary use on incidental receptive and productive vocabulary acquisition through reading [Master's Thesis, the American University in Cairo]. AUC Knowledge Fountain. https://fount.aucegypt.edu/etds/1154
19. Cao, Z. (2013). The effects of tasks on the learning of lexical bundles by Chinese EFL learners. *Theory and Practice in Language Studies*, 3(6), 957-962. https://doi.org/10.4304/tpls.3.6.957-962
20. Touti, M. A. E. (2013). Task-type based vocabulary instruction an impact on incidental word retention. *World Applied Sciences Journal*, 22(12), 1739-1744. https://doi.org/10.5829/idosi.wasj.2013.22.12.432
21. Mármol, G. A., & Sánchez-Lafuente, Á. A. (2013). The involvement load hypothesis: Its effect on vocabulary learning in primary education. *Revista Espanola de Linguistica Aplicada*, 26, 11-24.
22. Rouhi, A., & Mohebbi, H. (2013). Glosses, spatial intelligence, and L2 vocabulary learning in multimedia context. *3L: Language, Linguistics, Literature*, 19(2), 75-87.
23. Tajeddin, Z., & Daraee, D. (2013). Vocabulary acquisition through written input: Effects of form-focused, message-oriented, and comprehension tasks. *The Electronic Journal for English as a Second Language*, 16(4).
24. Jahangiri, K., & Abilipour, I. (2014). Effects of collaboration and exercise type on incidental vocabulary learning evidence against involvement load hypothesis. *Procedia Social and Behavioral Sciences Proceedings of the International Conference on Current Trends in Elt*, Amsterdam.
25. Li, J. (2014). Effect of task-induced online learning behavior on incidental vocabulary acquisition by Chinese learners - Revisiting involvement load hypothesis. *Theory and Practice in Language Studies,* 4(7), 1385-1394. https://doi.org/10.4304/tpls.4.7.1385-1394
26. Sarbazi, M. R. (2014). Involvement load hypothesis: Recalling unfamiliar words meaning by adults across genders *Proceedings of the International Conference on Current Trends in Elt*, Amsterdam.

27. Bao, G. (2015). Task type effects on English as a Foreign Language learners' acquisition of receptive and productive vocabulary knowledge. *System*, 53, 84-95. https://doi.org/10.1016/j.system.2015.07.006
28. Pourakbari, A. A., & Biria, R. (2015). Efficacy of task-induced involvement in incidental lexical development of Iranian senior EFL students. *English Language Teaching*, 8(5), 122-131. https://doi.org/10.5539/elt.v8n5p122
29. Soleimani, H., & Rahmanian, M. (2015). Vocabulary acquisition and task effectiveness in involvement load hypothesis: A case in Iran. *International Journal of Applied Linguistics and English Literature*, 4(5), 198-205. https://doi.org/10.7575/aiac.ijalel.v.4n.5p.19
30. Wang, J. H. T. (2015). Task-induced involvement load: The accumulation effects on vocabulary acquisition. *Chinese Journal of Applied Linguistics*, 38(2), 150-165. https://doi.org/10.1515/cjal-2015-0009
31. Ansarin, A. A., & Bayazidi, A. (2016). Task type and incidental L2 vocabulary learning: Repetition versus task involvement load. *Southern African Linguistics and Applied Language Studies*, *34*(2), 135-146. https://doi.org/10.2989/16073614.2016.1201774
32. Hu, H. C. M., & Nassaji, H. (2016). Effective vocabulary learning tasks: Involvement Load Hypothesis versus Technique Feature Analysis. *System*, 56, 28-39. https://doi.org/10.1016/j.system.2015.11.001
33. Tang, C., & Treffers-Daller, J. (2016). Assessing incidental vocabulary learning by Chinese EFL learners: a test of the involvement load hypothesis. In G. Yu & Y. Jin (Eds.), *Assessing Chinese Learners of English: Language Constructs, Consequences and Conundrums* (pp. 121-149). Palgrave Macmillan. http://dx.doi.org/10.1057/9781137449788
34. Zou, D. (2016). Comparing dictionary-induced vocabulary learning and inferencing in the context of reading. *Lexikos*, 26, 372-390.
35. Alharbi, R. M. (2017). Acquisition of lexical collocations: A corpus-assisted contrastive analysis and translation approach (Publication No. 2307366148) [Doctoral dissertation, Newcastle University]. ProQuest Dissertations and Theses Global.
36. Dowswell, K. (2017). Developing students' vocabulary knowledge in content subjects: A computational linguistic approach. Iaria Xps Press.
37. Lee, S., & Pulido, D. (2017). The impact of topic interest, L2 proficiency, and gender on EFL incidental vocabulary acquisition through reading. *Language Teaching Research*, *21*(1), 118-135. https://doi.org/10.1177/1362168816637381
38. Teng, F. M. (2017). Investigating task-induced involvement load and vocabulary learning from the perspective of metacognition. *Pertanika Journal of Social Sciences and Humanities,* 25(4), 1753-1764.
39. Qin, C., & Teng, F. (2017). Assessing the correlation between task-induced involvement load, word learning, and learners' regulatory ability. *Chinese Journal of Applied Linguistics*, 40(3), 261-280. https://doi.org/DOI:10.1515/cjal-2017-0015
40. Rassaei, E. (2017). Effects of three forms of reading-based output activity on L2 vocabulary learning. *Language Teaching Research*, 21(1), 76-95. https://doi.org/10.1177/1362168815606160
41. Sauer, B. M. (2016). The effects of different types of textual input enhancements on incidental and intentional vocabulary learning from

reading (Publication No. 2230937984) [Doctoral dissertation, King's College London]. ProQuest Dissertations and Theses Global.
42. Snoder, P. (2017). Improving English learners' productive collocation knowledge: The effects of involvement load, spacing, and intentionality. *TESL Canada Journal*, 34(3), 140-164. https://doi.org/10.18806/tesl.v34i3.1277
43. Tahmasbi, M., & Farvardin, M. T. (2017). Probing the effects of task types on EFL learners' receptive and productive vocabulary knowledge: The case of involvement load hypothesis. *SAGE Open*, *7*(3), 1-10. https://doi.org/10.1177/2158244017730596
44. Teng, F. (2017). The effects of task-induced involvement load on word learning and confidence judgments mediated by knowledge and regulation of cognition. *Educational Sciences: Theory & Practice*, *17*(3), 791-808. https://doi.org/10.12738/estp.2017.3.0167
45. Tong, S. (2017). Effects of glosses and involvement loads on incidental vocabulary acquisition. *Francis Acad Press*
46. Yang, Y. L., Shintani, N., Li, S. F., & Zhang, Y. Y. (2017). The effectiveness of post-reading word-focused activities and their associations with working memory. *System*, 70, 38-49. https://doi.org/10.1016/j.system.2017.09.012
47. Zou, D. (2017). Vocabulary acquisition through cloze exercises, sentence-writing and composition-writing: Extending the evaluation component of the involvement load hypothesis. *Language Teaching Research*, *21*(1), 54-75. https://doi.org/10.1177/1362168816652418
48. Alkhudiry, R. (2018). Exploring the relationship between vocabulary knowledge and reading comprehension in L1 Arabic learners of English (Publication No. 2189022640) [Doctoral dissertation, University of Reading]. ProQuest Dissertations and Theses Global.
49. Gohar, M. J., Rahmanian, M., & Soleimani, H. (2018). Technique feature analysis or involvement load hypothesis: estimating their predictive power in vocabulary learning. *Journal of Psycholinguistic Research*, *47*(4), 859-869. https://doi.org/10.1007/s10936-018-9568-5
50. Huang, S. Y. (2018). A verification of involvement load hypothesis on chinese adult English learners. *International Journal of English Linguistics*, *8*(5), 125-134. https://doi.org/10.5539/ijel.v8n5p125
51. Ong, K. K. W., & Zhang, L. J. (2018). The effects of code-switched reading tasks on late-bilingual EFL learners' vocabulary recall, retention and retrieval. *System*, *72*, 13-22. https://doi.org/10.1016/j.system.2017.10.008
52. Silva, B., & Otwinowska, A. (2018). Vocabulary acquisition and young learners: Different tasks, similar involvement loads. *International Review of Applied Linguistics in Language Teaching*, *56*(2), 205-229. https://doi.org/10.1515/iral-2016-0097
53. Alanazi, Z. (2019). The effects of a sentence completion task vs. A sentence generation task on vocabulary learning: An exploratory study. *Linguistics Journal*, *13*(1), 147-172.
54. Alavinia, P., & Rahimi, H. (2019). Task types effects and task involvement load on vocabulary learning of EFL learners. *International Journal of Instruction*, *12*(1), 1501-1516. https://doi.org/10.29333/iji.2019.12196a
55. Bao, G. (2019). Comparing input and output tasks in EFL learners' vocabulary acquisition. *TESOL International Journal*, *14*(1), 1-12.
56. Dai, Y., Wu, Z., & Xu, H. (2019). The effect of types of dictionary presentation on the retention of metaphorical collocations: Involvement

load hypothesis vs. cognitive load theory. *International Journal of Lexicography*, *32*(4), 411-431. https://doi.org/10.1093/ijl/ecz010
57. Kıvrak, C. (2019). The effects of task involvement load and input type on foreign language vocabulary learning (Publication No. 2499374271) [Master Thesis, Marmara University]. ProQuest Dissertations and Theses Global.
58. Nguyen, C. D., & Boers, F. (2019). The effect of content retelling on vocabulary uptake from a TED talk. *TESOL Quarterly*, 53(1), 5-29. https://doi.org/10.1002/tesq.441
59. Alahmadi, A., & Foltz, A. (2020). Exploring the effect of lexical inferencing and dictionary consultation on undergraduate EFL students' vocabulary acquisition. *PLoS ONE*, *15*(7), 25. https://doi.org/10.1371/journal.pone.0236798
60. Alarjani, F. A. (2020). Involvement load hypothesis and the retention of word meaning among saudi EFL learners. *Asiatic*, 14(1), 160-173.
61. Arabiana, E. F. S., Malifer, D. A. E. A., & Betonio, H. R. (2020). Video cartoons and task-induced involvement: effects to pupils' l2 incidental literacy acquisition. *English Language Teaching Educational Journa*l, 3(2), 151-162.
62. Chiu, H. H., & Chen, C. F. (2020). A comparison of EFL fifth graders' vocabulary acquisition through skype videoconferencing and face-to-face picture book storytelling. *Journal of Language and Education,* 6(2), 91-105. https://doi.org/https://doi.org/10.17323/jle.2020.10082
63. Danilina, S., & Shabunina, V. (2020). Intentional vs incidental ESP vocabulary acquisition by political science students. *Advanced Education* (16), 18-27.
64. Dongho, K. (2020). The effects of task-induced involvement on l2 academic word acquisition in Korea. *Korean Journal of English Language and Linguistics*, 2020 (20), 141-156. https://doi.org/10.15738/kjell.20.202005.141
65. Kaivanpanah, S., Alavi, S. M., & Ravandpour, A. (2020). The effect of input-based and output-based tasks with different and identical involvement loads on Iranian EFL learners' incidental vocabulary learning. *Cogent Psychology*, 7(1), 16, Article 1731223. https://doi.org/10.1080/23311908.2020.1731223
66. Kamali, M., Behjat, F., & Bagheri, M. S. (2020). Examining the effects of oral reproduction and summary writing vocabulary tasks on L2 word learning: Technique feature analysis on trial. *Cogent Education*, 7(1), 25. https://doi.org/10.1080/2331186x.2020.1795966
67. Namaziandost, E., Hosseini, E., & Utomo, D. W. (2020). A comparative effect of high involvement load versus lack of involvement load on vocabulary learning among Iranian sophomore EFL learners. *Cogent Arts & Humanities*, *7*(1), 15. https://doi.org/10.1080/23311983.2020.1715525
68. Rassaei, E. (2020). Effects of mobile-mediated dynamic and nondynamic glosses on L2 vocabulary learning: A sociocultural perspective. *Modern Language Journal*, 104(1), 284-303. https://doi.org/10.1111/modl.12629
69. Xu, Y., & Zhang, J. (2020). Chinese compound word inference through context and word-internal cues. *Language Teaching Research*, 1-25. https://doi.org/https://doi.org/10.1177/1362168820905811

70. Yang, Y., & Cao, X. (2020). Effects of task involvement load on L2 vocabulary acquisition and their association with language aptitude. *Asia-Pacific Education Researcher.* https://doi.org/10.1007/s40299-020-00528-8
71. Alcaraz-Marmol, G. (2021). Glossing and L2 vocabulary learning through dynamic instruction in the context of primary education. *Revista De Linguistica Y Lenguas Aplicadas*, *16*, 1-10. https://doi.org/10.4995/rlyla.2020.14691
72. Ansarin, A. A., & Khabbazi, S. K. (2021). Task-induced involvement load and working memory: Effects on active and passive vocabulary knowledge of EFL learners in a multimedia learning environment. *Eurasian Journal of Applied Linguistics*, 7(1), 277-302. https://doi.org/10.32601/ejal.911288
73. Danilina, S. (2021). Collocational knowledge uptake by university students under online learning. *English Studies at Nbu*, 7(1), 97-117. https://doi.org/https://doi.org/10.33919/esnbu.21.1.7
74. Hanks, L. (2021). The effect of vocabulary task type on student semantic and orthographic retention] (Publication No. 2531525944) [Master thesis, Northeastern Illinois University]. ProQuest Dissertations and Theses Global.
75. San Mateo-Valdehita, A., & de Diego, C. C. (2021). Receptive and productive vocabulary acquisition: effectiveness of three types of tasks. Results from French students of Spanish as second language. *Onomazein* (51), 36-56. https://doi.org/10.7764/onomazein.51.05
76. Silva, B. B., Kutyłowska, K., & Otwinowska, A. (2021). Learning academic words through writing sentences and compositions: Any signs of an increase in cognitive load? *Language Teaching Research.* https://doi.org/10.1177/13621688211020421
77. Taheri, S., & Golandouz, G. R. (2021). The effect of task type on EFL learners' acquisition and retention of vocabulary: an evaluation of the involvement load hypothesis. *Cogent Education*, 8(1), 21. https://doi.org/10.1080/2331186x.2021.1915226
78. Teng, M. F., & Zhang, D. (2021). Task-induced involvement load, vocabulary learning in a foreign language, and their association with metacognition. *Language Teaching Research*. https://doi.org/10.1177/13621688211008798

Supplementary Table S5. Specific scales for rating studies

WoE Scale	Level	Description
Soundness	High	The research methods and results are described clearly and in detail. The authors explain their results/findings clearly and transparently.
	Medium	The research methods and results are described satisfactorily. The results explained by the authors are generally satisfactory.
	Low	The description of research methods and results is vague. The results of the study explained by the authors are confusing
Appropriateness	High	The methodology of the selected study is consistent with the review questions.
	Medium	The methodology of the selected study is partially consistent with the review questions.
	Low	The methodology of the selected study is not consistent with the review questions.
Relevance	High	The selected studies provide sufficient information to answer the review questions.
	Medium	The selected studies provide some information that can be combined and/or synthesized to answer the review questions.
	Low	The selected studies do not provide any information that could be combined and/ or synthesized to answer the review question.

Supplementary Figure S2. High Frequency Task Types and the ILH Support in English as the Target Language Studies

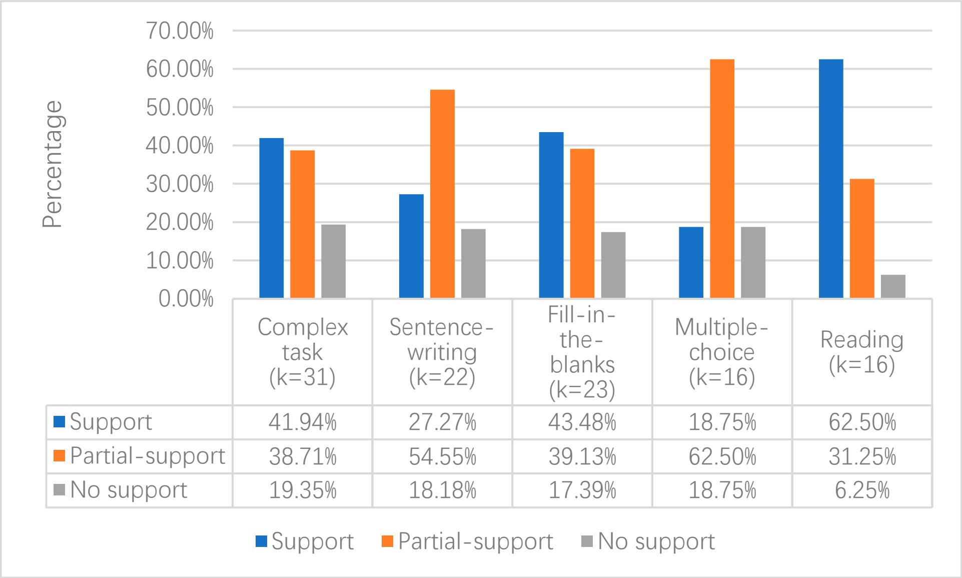

Supplementary Figure S3. Medium frequency task types and the ILH support in English as the target language studies

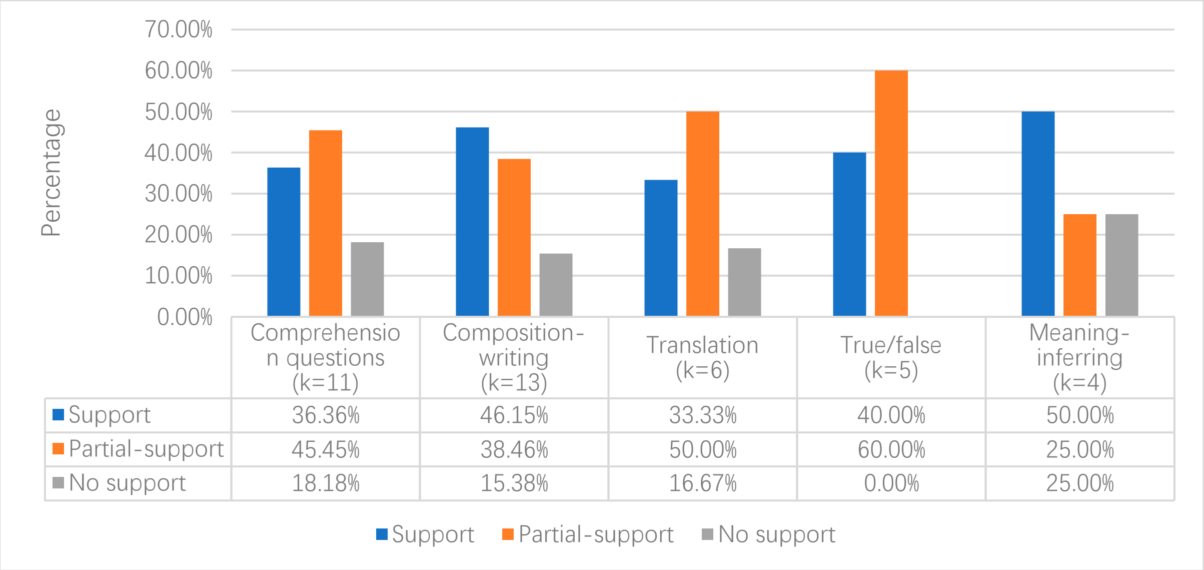

	Comprehension questions (k=11)	Composition-writing (k=13)	Translation (k=6)	True/false (k=5)	Meaning-inferring (k=4)
Support	36.36%	46.15%	33.33%	40.00%	50.00%
Partial-support	45.45%	38.46%	50.00%	60.00%	25.00%
No support	18.18%	15.38%	16.67%	0.00%	25.00%

Supplementary Figure S4. Low frequency task types and the ILH support in English as the target language studies

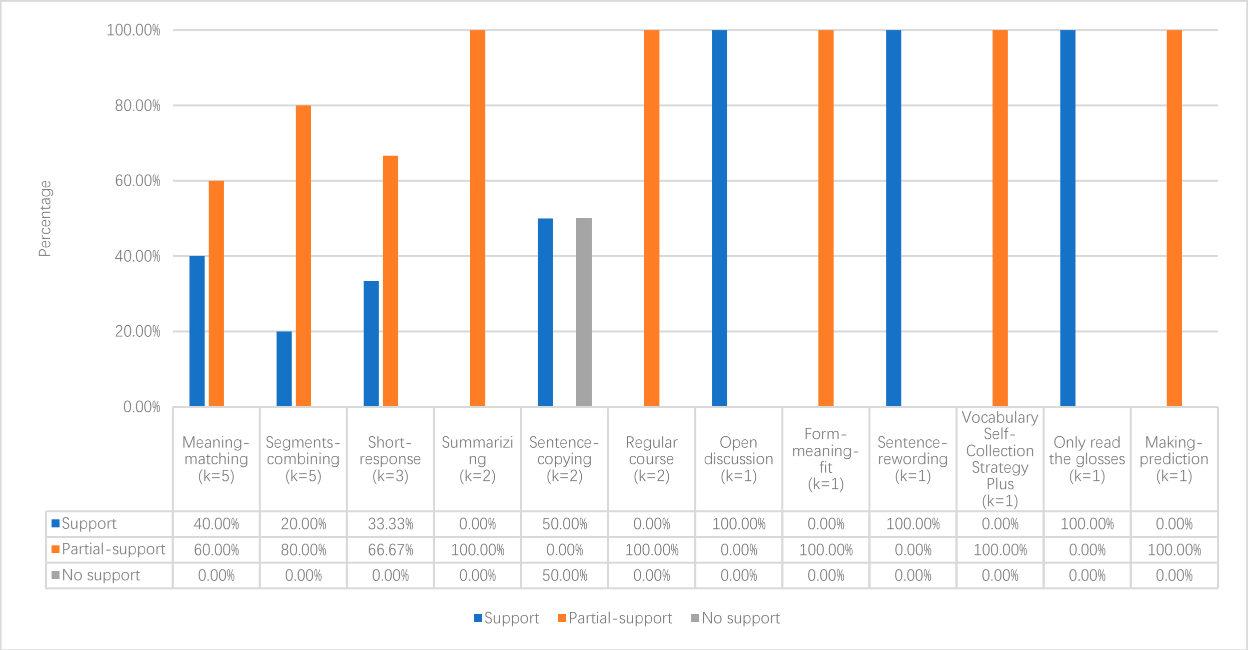

Supplementary Figure S5. High frequency task types and the ILH support in studies (any language as the target language)

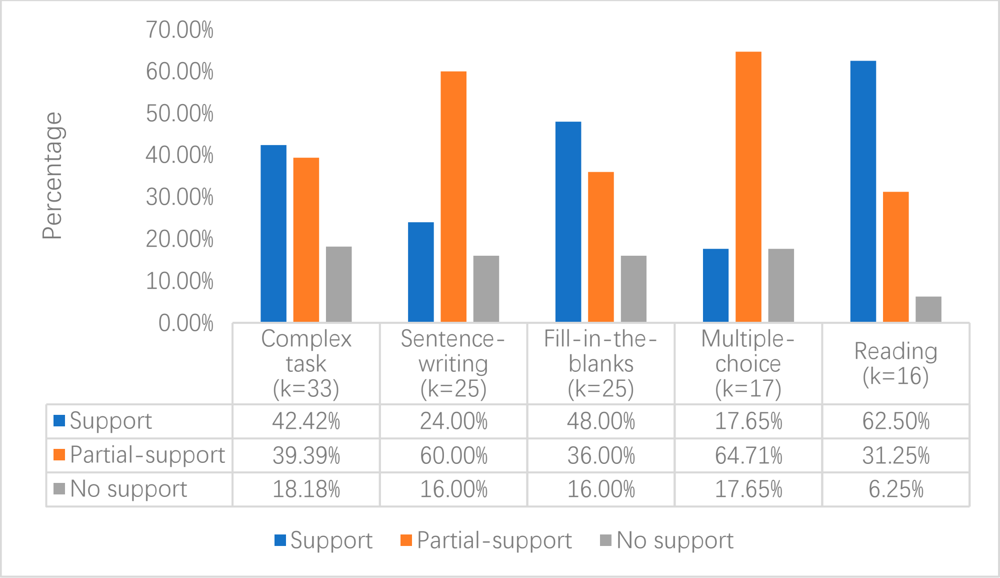

Supplementary Figure S6. Medium frequency task types and the ILH support in studies (any language as the target language)

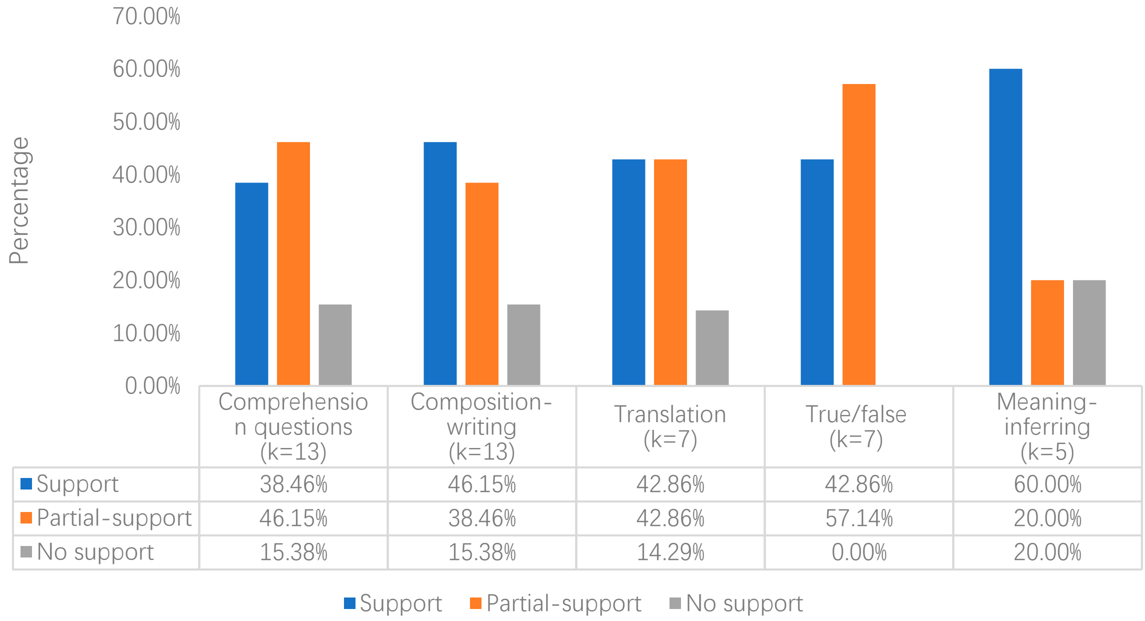

	Comprehension questions (k=13)	Composition-writing (k=13)	Translation (k=7)	True/false (k=7)	Meaning-inferring (k=5)
Support	38.46%	46.15%	42.86%	42.86%	60.00%
Partial-support	46.15%	38.46%	42.86%	57.14%	20.00%
No support	15.38%	15.38%	14.29%	0.00%	20.00%

Supplementary Figure S7. Low frequency task types and the ILH support in studies (any language as the target language)

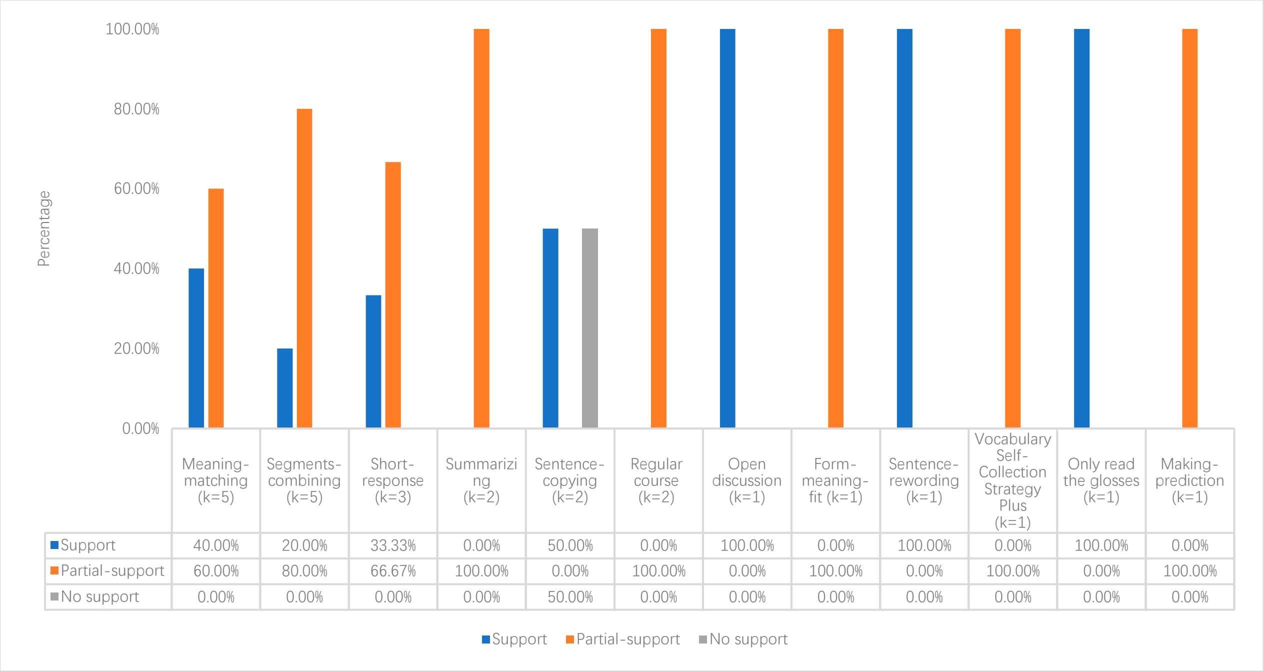

	Meaning-matching (k=5)	Segments-combining (k=5)	Short-response (k=3)	Summarizing (k=2)	Sentence-copying (k=2)	Regular course (k=2)	Open discussion (k=1)	Form-meaning-fit (k=1)	Sentence-rewording (k=1)	Vocabulary Self-Collection Strategy Plus (k=1)	Only read the glosses (k=1)	Making-prediction (k=1)
Support	40.00%	20.00%	33.33%	0.00%	50.00%	0.00%	100.00%	0.00%	100.00%	0.00%	100.00%	0.00%
Partial-support	60.00%	80.00%	66.67%	100.00%	0.00%	100.00%	0.00%	100.00%	0.00%	100.00%	0.00%	100.00%
No support	0.00%	0.00%	0.00%	0.00%	50.00%	0.00%	0.00%	0.00%	0.00%	0.00%	0.00%	0.00%

Supplementary Table S6. Prevalence of task types (language other than English as the target language)

Task type	Tasks	
	n	%
Complex task	5	23.81
Sentence-writing	3	14.29
Fill-in-the-blanks	3	14.29
Comprehension questions	3	14.29
Translation	2	9.52
True/false	2	9.52
Meaning-inferring	2	9.52
Multiple-choice	1	4.76
Total	21	100.00

Note: The task types were given in descending order.

In general, six empirical studies had used the ILH as a framework to study non-English vocabulary learning. The targeted languages were Spanish, Italian, German, and Chinese. A total of 21 different tasks were scattered across eight types (See Table S6).

Similar to English as the target language studies, the most common task type of other language as target language studies was the complex task type, combining several individual tasks into one task. A total of five different complex tasks were found in two studies. One study showed that complex tasks designed for Italian vocabulary learning provided support for the predictability of the ILH (Rukholm, 2011). The other study found that complex tasks designed for Spanish vocabulary learning provided only partial support for the predictability of the ILH (Keating, 2008).

A total of three different sentence-writing tasks were scattered among three studies. All of the three studies showed that sentence-writing tasks provided at least some support for the predictability of the ILH. In Rukholm (2011)'s study of Italian vocabulary learning, the sentence writing task provided support for the ILH. Two other studies provided partial support

for the predictability of the ILH in Spanish vocabulary learning (Keating, 2008; San Mateo-Valdehita & de Diego, 2021). Regarding fill-in-the-blanks task type, a total of three different tasks were found in two studies. Similarly, the tasks designed for Italian vocabulary learning provided support for the ILH (Rukholm, 2011), and the task designed for Spanish vocabulary learning provided partial support for the ILH (San Mateo-Valdehita & de Diego, 2021). Three different comprehension questions tasks were found in two studies. The study which investigated German vocabulary learning showed partial support for the ILH (Peters, 2007). The study which investigated Italian vocabulary learning showed support of the ILH (Rukholm, 2011).

Two different translation tasks were compared in one study which focused on Spanish vocabulary learning. The results corroborated the assumption of the ILH that that different specific tasks with the same IL have the same vocabulary learning effect when other factors are equal (Laufer & Hulstijn, 2001). Two different true/false tasks were found in two studies. Similar to the complex tasks, the true/false task designed for Italian vocabulary learning provided support for the ILH, while the true/false task designed for Spanish vocabulary learning partially supported the ILH (Keating, 2008; Rukholm, 2011). Two different meaning-inferring tasks were compared in one study which focused on Chinese vocabulary learning (Xu & Zhang, 2020). In this study, researchers compare two meaning-inferring tasks with different ILs. The meaning-inferring task with higher IL was found more effective in Chinese vocabulary learning than the meaning-inferring task with lower IL. The results corroborated the second assumption of the ILH that "Other factors being equal, words which are processed with higher involvement load will be retained better than words which are processed with lower

involvement load” (Laufer & Hulstijn, 2001, p. 15). Only one multiple-choice task was found in one study. The results showed partial support for the ILH in the context of Spanish vocabulary learning (San Mateo-Valdehita & de Diego, 2021).

References:

Rukholm, V. N. (2011). *Facilitating lexical acquisition in beginner learners of Italian through popular song* (Publication No. 1323346428) [Doctoral dissertation, University of Toronto]. ProQuest Dissertations and Theses Global.

Keating, G. D. (2008). Task effectiveness and word learning in a second language: The involvement load hypothesis on trial. Language Teaching Research, 12(3), 365-386. https://doi.org/10.1177/1362168808089922

San Mateo-Valdehita, A., & de Diego, C. C. (2021). Receptive and productive vocabulary acquisition: effectiveness of three types of tasks. Results from French students of Spanish as second language. Onomazein (51), 36-56. https://doi.org/10.7764/onomazein.51.05

Peters, E. (2007). Manipulating L2 learners' online dictionary use and its effect on L2 word retention. Language Learning & Technology, 11(2), 36-58.

Laufer, B., & Hulstijn, J. (2001). Incidental vocabulary acquisition in a second language: The construct of task-induced involvement [Article]. Applied Linguistics, 22(1), 1-26. https://doi.org/10.1093/applin/22.1.1

Xu, Y., & Zhang, J. (2020). Chinese compound word inference through context and word-internal cues. Language Teaching Research, 1-25. https://doi.org/https://doi.org/10.1177/1362168820905811
